# Patient‐reported health‐related quality of life in previously untreated chronic lymphocytic leukaemia: Results from the randomised phase 3 FLAIR trial comparing ibrutinib–rituximab versus fludarabine–cyclophosphamide–rituximab

**DOI:** 10.1111/bjh.70416

**Published:** 2026-03-30

**Authors:** David J. Allsup, David A. Cairns, E. Faye Samy, Lelia Duley, Adrian Bloor, Abraham Varghese, David Meads, Bryony Dawkins, Sean Girvan, Dena R. Howard, Anna Hockaday, Julia M. Brown, Sharon Jackson, Natasha Greatorex, Phoebe Templeton, Michael Tupper, David Phillips, Sonam Yaqub, Di Mortimer, David Stones, Piers E. M. Patten, Andrew Rawstron, Peter Hillmen, Talha Munir

**Affiliations:** ^1^ Centre for Biomedicine, Hull York Medical School University of Hull Hull UK; ^2^ Leeds Cancer Research UK Clinical Trials Unit, Leeds Institute of Clinical Trials Research University of Leeds Leeds UK; ^3^ Frontier Science Scotland Kingussie UK; ^4^ CLL Support Chippenham UK; ^5^ The Christie Hospital NHS Foundation Trust Manchester UK; ^6^ Leeds Cancer Centre, Leeds Teaching Hospitals Trust Leeds UK; ^7^ Academic Unit of Health Economics, Leeds Institute of Health Sciences University of Leeds Leeds UK; ^8^ Comprehensive Cancer Centre King's College London London UK; ^9^ Department of Haematology King's College Hospital London UK; ^10^ Haematological Malignancy Diagnostic Service (HMDS), Leeds Cancer Centre Leeds Teaching Hospitals Trust Leeds UK

**Keywords:** Bruton tyrosine kinase inhibitor, chemoimmunotherapy, chronic lymphocytic leukaemia, health‐related quality of life, randomised controlled trial

## Abstract

Front‐Line therapy in CLL: Assessment of Ibrutinib‐containing Regimens (FLAIR) demonstrated improved progression‐free survival for ibrutinib and rituximab (IR) compared with fludarabine, cyclophosphamide and rituximab (FCR) in previously untreated chronic lymphocytic leukaemia (CLL). This report presents the secondary end‐point of health‐related quality of life (HR‐QoL). FLAIR was a phase 3, open‐label, randomised trial across 101 hospitals. Eligible patients were aged 18–75 years, World Health Organization performance status (PS) ≤2, requiring treatment; those with >20% 17p deletion were excluded. IR was administered for up to 6 years and FCR for six cycles. Participants completed European Organisation for Research and Treatment of Cancer Quality of Life C30 Questionnaire (EORTC‐QLQ‐C30), QLQ CLL Module (QLQ‐CLL16), three‐level EQ‐5D (EQ‐5D‐3L) and EQ5D visual analogue (EQ‐VAS) at baseline and follow‐up. Function and symptom trajectories were analysed using repeated‐measures multilevel regression. 84.4% of participants completed baseline questionnaires and subsequent compliance was 67.6%–83.5%. Median age was 63 years; most participants were white and male. HR‐QoL trajectories were similar. FCR recipients had worse scores at end of treatment but recovered thereafter. By 48 months, more FCR‐treated participants showed meaningful improvements in several scales. Statistically significant differences (*p* < 0.05) favoured IR for physical, role and social function; emotional function favoured FCR. Diarrhoea was more common with IR; fatigue and dyspnoea were more common with FCR, though differences did not exceed minimally important thresholds. Overall, scales were comparable between treatment groups, indicating that continuous IR does not compromise HR‐QoL.

## INTRODUCTION

Chronic lymphocytic leukaemia (CLL) is the second most common haematological malignancy and affects six adults per 100 000.^1^ Historically, front‐line CLL treatment was based upon chemoimmunotherapeutic regimens, such as fludarabine, cyclophosphamide plus rituximab (FCR). FCR was primarily deployed in fitter patients considered able to tolerate more intensive therapy. The development of Bruton tyrosine kinase inhibitors (BTKis) such as ibrutinib, acalabrutinib and zanubrutinib and small‐molecule inhibitors of the B‐cell lymphoma 2 molecule (Bcl‐2), such as venetoclax has improved the outlook for those with CLL. Such improvements include prolonged progression‐free^2^, ^3^, ^4^ and overall survival (PFS and OS)^5^ with increased probability of attaining undetectable measurable residual disease (uMRD).^6^, ^7^, ^8^ The initial regulatory approvals of BTKi were based upon clinical trials in which ibrutinib was administered until disease progression in comparison to low‐intensity chemoimmunotherapy. This shifted the CLL treatment paradigm from fixed duration to continuous therapy. Fixed duration doublet combinations of either anti‐CD20 monoclonal antibodies with a BTKi or a combination of BTKis and Bcl‐2 inhibitors have subsequently been developed. The combinations of venetoclax with obinutuzuamb (an anti‐CD20 monoclonal antibody) and ibrutinib with venetoclax are currently the most studied doublet regimens with associated survival and MRD advantages over historic chemotherapy therapies. There is strong evidence for the effectiveness of novel agents given as both continuous,^3^, ^9^ fixed duration^4^, ^6^ and MRD‐guided regimens.^5^

The impact of novel treatment combinations or strategies on patient‐reported health‐related quality of life (HR‐QoL) is important to regulators,^10^ health‐care funders^11^ and patients (as shown in the UK CLL Support national survey^12^). Persons with treatment‐requiring CLL report negative impacts of their disease on fatigue, sleep, role function and global health status as compared to the general population.^13^ Studies of HR‐QoL have shown improvements associated with therapy in previously untreated, comorbid, older populations^14^ and in those with relapsed disease.^15^ However, the number of reports describing the long‐term impact of treatment and disease on quality of life in the younger, non‐comorbid CLL population is limited.

The Front‐Line therapy in CLL: Assessment of Ibrutinib‐containing Regimens (FLAIR) trial compared the efficacy and toxicity of ibrutinib combined with rituximab (IR) with FCR in previously untreated patients with CLL who required treatment and were fit for fludarabine‐based combination chemoimmunotherapy. In this report, we describe the secondary end‐point of HR‐QoL after the recent report of the first formal interim analysis of the primary end‐point of PFS.^9^

## METHODS

### Study design and participants

FLAIR is a phase 3, open‐label, randomised, controlled trial in persons with previously untreated CLL. Inclusion criteria and exclusion criteria are available in Supporting Information. The trial recruited from 101 National Health Service hospitals in England, Wales, Scotland and Northern Ireland (Table S1). All participants provided written informed consent. This study was approved by the national ethics review board (National Research Ethics Service, London, UK) and the competent regulatory authority (Medicines and Healthcare Products Regulatory Agency, London, UK). This study is International Standard Registered Clinical/Social Study Number (ISRCTN) and clinicaltrialsregister.eu registered (ISRCTN01844152 and 2013‐001944‐76).

### Randomisation and masking

Participants were randomly assigned (1:1) to treatment with either IR or FCR. A computer‐generated minimisation algorithm with a random element was used to avoid chance imbalances in three variables determined at trial entry: Binet stage (Progressive A or B, C), age (≤65 years, >65 years), sex (male, female) and hospital (Table S1).

### Procedures

FCR was delivered orally every 28 days for a total of six cycles in the absence of disease progression or toxicity requiring cessation. Ibrutinib was administered with rituximab scheduled like FCR, and then, ibrutinib was delivered in the absence of disease progression or toxicity requiring cessation for either 6 years, until MRD stopping rules were reached.

Participants completed three validated QoL questionnaires: the European Organisation for Research and Treatment of Cancer Quality of Life C30 Questionnaire (EORTC‐QLQ‐C30),^16^ the CLL‐specific module, EORTC‐QLQ‐CLL16 (QLQ CLL Module) and the EuroQoL 5D‐3L.^17^ Questionnaires were completed at the following time points: prior to randomisation; at the end of treatment with rituximab (FCR/IR); and then every 6 months from 12 months after randomisation until 7 years post‐randomisation, or until treatment for progressive disease, whichever occurred earliest.

### Outcomes

The primary end‐point of FLAIR was PFS, defined as the time from randomisation to progressive disease or death (from any cause). Participants without an event were censored at the time of last follow‐up. Secondary end‐points of the trial included OS, uMRD in the bone marrow and peripheral blood, clinical responses to therapy according to iwCLL criteria, safety and toxicity as graded by National Cancer Institute Common Terminology Criteria for Adverse Events (CTCAE) V5.0, HR‐QoL and cost‐effectiveness assessed using the social functioning‐12 and EuroQol 5 dimensions (EQ‐5D) to produce quality‐adjusted life‐years (QALYs).

### Statistical analysis

QoL analysis focussed on the patient‐reported outcome (PRO) intention‐to‐treat (ITT) population which consisted of participants who gave consent to complete QoL questionnaires and who returned the baseline questionnaire.

Mean baseline HR‐QoL scores from FLAIR were compared with post hoc selected benchmark values from the UK reference populations. EORTC‐QLQ‐C30 reference scores^18^ were reweighted by age distribution and sex of FLAIR participants (*N* = 1026). EQ‐5D reference scores^19^ were from the UK general population (*N* = 3395).

Least square means and 95% confidence intervals were estimated for each QoL scale with linear models adjusted for the baseline score.

A multilevel repeated measures model was used to compare groups. This was chosen to allow for a wide variety of correlation structures described in Supporting Information. This model does not require complete data from all participants, which leads to more appropriate estimates of the treatment effect and standard errors. This type of analysis requires at least one valid score per participant to be included in the model. We excluded any participant with an omitted baseline score.

The proportion of participants with a meaningful improvement, meaningful decline or stable QoL compared with baseline was estimated for each subscale.^20^, ^21^ Improvement and decline was defined as a difference of ≥10 points compared with baseline for the QLQ‐C30 and QLQ‐CLL16 and EQ5D visual analogue (EQ‐VAS) of the EQ‐5D, and 0.1 points for the EQ‐5D. A participant's QoL was considered ‘stable’ otherwise.

The data cut‐off date for this analysis was 24 May 2021. All reported p values are two‐sided and considered significant at the 5% level. SAS was employed (version 9.4, SAS Institute, Cary NC) for statistical analyses.

## RESULTS

Seven hundred and seventy‐one patients with CLL were randomised between 19 September 2014 and 19 July 2018.

Of 771 participants, 651 (84.4%) completed baseline PROs (Figure S1). Compliance with completing the questionnaires was 68.0%–82.7% for QLQ‐C30, 67.6%–82.1% for QLQ‐CLL16 and 68.3%–83.5% for EQ‐5D up to month 48 (Tables S3–S5). Item‐level responses were rarely omitted in returned forms (<10%) (data not shown), leading to a moderate rate of missingness at the scale level in EQ5D Utility Index (3.6%–13.0%) (Table S3) and a low rate of missingness at the scale level for QLQ–C30 and QLQ–CLL16 (<2%) (Tables S4 and S5).

The PRO ITT population for this analysis comprised 651 participants with 329 participants randomly assigned to the IR group and 322 participants assigned to the FCR group. The characteristics of the FLAIR ITT population have been reported previously and are summarised in Appendix S1 (Table S2). There were no major differences in the characteristics of those in the PRO ITT population (Table 1) and those in the FLAIR ITT population in terms of age, sex, ethnicity, performance status and duration of CLL (Table S2). The median age of participants was 63 years (range 27–76); 174 (27%) of participants were female; and 620 (95%) participants were White, 8 (1%) Black and 3 (<1%) Asian. Demographics and clinical characteristics were generally well balanced between treatment groups, except for World Health Organization performance status (PS, 208 [63%] of 329 participants in the IR group were PS 0 vs. 222 [69%] of 322 participants in the FCR group). Most participants were Binet Stage Progressive A or B (186 [57%] of 329 assigned to IR vs. 184 [57%] of 322 receiving FCR), with a median duration of CLL diagnosis to FLAIR randomisation of 24 months (range 0–219).

**TABLE 1:** Baseline characteristics by randomised group in the QoL population including mean baseline PRO scores.

	FCR (*n* = 322)	IR (*n* = 329)	Reference populations
Gender			
Male	232 (72.0%)	245 (74.5%)	
Female	90 (28.0%)	84 (25.5%)	
Age			
Median (range)	62.9 (39.2, 75.7)	63.5 (27.0, 74.2)	
≤65 years	214 (66.5%)	214 (65.0%)	
>65 years	108 (33.5%)	115 (35.0%)	
Binet stage			
Progressive A or B	184 (57.1%)	186 (56.5%)	
C	138 (42.9%)	143 (43.5%)	
Ethnicity			
White	307 (95.3%)	313 (95.1%)	
Mixed—White and Asian	0 (0.0%)	1 (0.3%)	
Other mixed background	0 (0.0%)	1 (0.3%)	
Asian—Indian	2 (0.6%)	0 (0.0%)	
Asian—Pakistani	1 (0.3%)	0 (0.0%)	
Black—Caribbean	2 (0.6%)	2 (0.6%)	
Black—African	2 (0.6%)	1 (0.3%)	
Other Black background	1 (0.3%)	0 (0.0%)	
Other ethnic group	2 (0.6%)	0 (0.0%)	
Not stated	5 (1.6%)	11 (3.3%)	
Duration of CLL (months)			
Mean (SD)	36.0 (36.5)	34.4 (36.5)	
Median (range)	24.2 (0.00, 162)	24.0 (0.00, 219)	
Missing	35	25	
WHO performance status			
0	222 (68.9%)	208 (63.2%)	
1	95 (29.5%)	108 (32.8%)	
2	4 (1.2%)	12 (3.6%)	
3	0 (0.0%)	0 (0.0%)	
4	0 (0.0%)	0 (0.0%)	
Missing	1 (0.3%)	1 (0.3%)	
Functioning scales			
Baseline EQ5D‐3L (*N* = 651)			
EQ5D‐3L Utility Index (mean (SD))	0.81 (0.23)	0.83 (0.22)	0.81
EQ‐VAS (mean (SD))	75.1 (19.0)	74.6 (18.6)	80.1
Baseline EORTC QLQ‐C30 (*N* = 651)			
Physical functioning (mean (SD))	83.0 (19.9)	82.6 (19.2)	80.6
Role functioning (mean (SD))	76.2 (30.0)	73.7 (29.0)	79.8
Emotional functioning (mean (SD))	75.8 (22.7)	78.0 (20.3)	77.1
Cognitive functioning (mean (SD))	82.0 (22.0)	84.0 (19.6)	83.5
Social functioning (mean (SD))	77.7 (28.5)	78.9 (26.3)	83.1
Global health status/QoL (mean (SD))	70.6 (20.9)	70.2 (20.7)	61.6
Symptom scales			
Baseline EORTC QLQ‐C30 (*N* = 651)			
Fatigue (mean (SD))	34.4 (25.8)	35.9 (26.0)	30.0
Nausea/vomiting (mean (SD))	4.97 (12.7)	4.66 (11.9)	3.8
Pain (mean (SD))	18.9 (27.4)	16.9 (23.2)	27.9
Dyspnoea (mean (SD))	23.0 (27.8)	21.7 (25.7)	18.8
Insomnia (mean (SD))	33.5 (32.2)	32.7 (30.3)	32.9
Appetite loss (mean (SD))	15.1 (24.8)	15.2 (25.2)	10.4
Constipation (mean (SD))	7.25 (17.5)	7.09 (16.8)	10.5
Diarrhoea (mean (SD))	11.4 (20.2)	8.00 (17.7)	7.4
Financial problems (mean (SD))	14.6 (28.5)	13.4 (27.0)	10.4
Baseline EORTC QLQ‐CLL16			
Fatigue scale (mean (SD))	35.3 (28.2)	37.4 (29.4)	
Treatment side effects scale (mean (SD))	16.8 (17.6)	15.1 (14.7)	
Disease effects scale (mean (SD))	21.2 (19.8)	20.3 (18.4)	
Infection scale (mean (SD))	14.0 (15.7)	14.9 (16.9)	
Social problems (mean (SD))	21.5 (29.6)	22.6 (29.4)	
Future health (mean (SD))	46.6 (30.6)	46.4 (29.1)	

*Note*: EORTC QLQ‐C30 reference scores were based on general population in United Kingdom (*N* = 1026) and reweighted by the age distributions and sex of FLAIR PRO ITT population^18^; EQ‐5D‐5L reference scores were from the UK general population (*N* = 3395).^19^

Abbreviations: CLL, chronic lymphocytic leukaemia; EORTC, European Organisation for Research and Treatment of Cancer; EQ‐5D, EuroQol 5 dimensions; EQ‐5D‐3L, three‐level EQ‐5D; EQ‐VAS, EQ5D visual analogue; FCR, fludarabine, cyclophosphamide and rituximab; GHS–QoL, global health status–quality of life; IR, ibrutinib and rituximab; PRO, patient‐reported outcome; QLQ‐C30, Quality of Life C30 Questionnaire; QLQ‐CLL16, QLQ CLL Module; QoL, quality of life; VAS, visual analogue scale; WHO, World Health Organization.

Baseline HR‐QoL scores for the FLAIR PRO ITT population were similar to those of reference populations across most scales (Table 1). For functioning scales, in EQ5D, the Utility Index was similar, but EQ‐VAS was lower in FLAIR participants than the reference population. For EORTC‐QLQ‐C30, Global health status/quality of life was higher in FLAIR participants than the reference population. For symptom scales, fatigue was higher in FLAIR participants than the reference population; pain was lower in FLAIR participants than the reference population; appetite loss was higher in FLAIR participants than the reference population.

**FIGURE 1:**
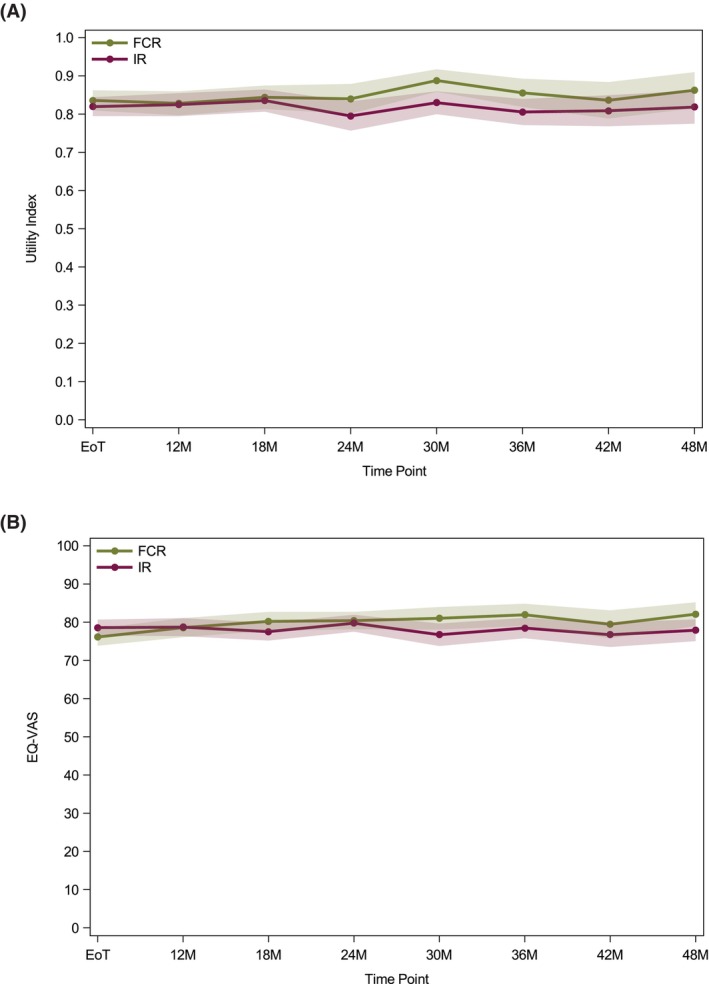
Mean scores adjusted for baseline for (A) EQ‐5D Utility Index and (B) EQ‐VAS. EQ‐VAS, EQ5D visual analogue; UI, EQ5D‐3L Utility Index.

**FIGURE 2:**
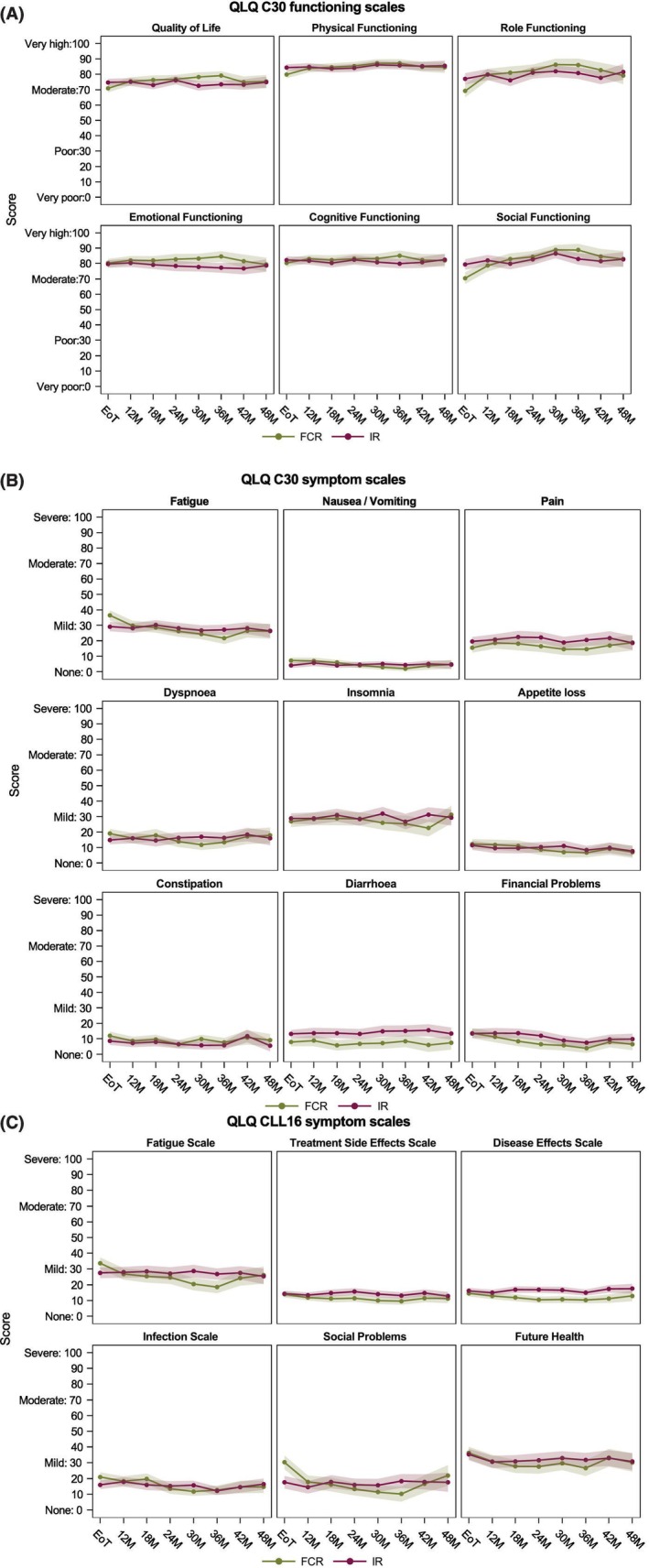
Mean scores adjusted for baseline for (A) EORTC QLQ C30 functioning scales, (B) EORTC QLQ C30 symptom scales and (C) EORTC QLQ CLL16 symptom scales. EORTC, European Organisation for Research and Treatment of Cancer; FCR, fludarabine, cyclophosphamide and rituximab; GHS–QoL, global health status–quality of life; IR, ibrutinib and rituximab; QLQ‐C30, Quality of Life C30 Questionnaire; QLQ‐CLL16, QLQ CLL Module.

Comparison of the IR and FCR groups in the period from the end of treatment did not show markedly different trajectories of quality of life as measured by the EQ‐5D Utility Index or EQ‐VAS when adjusted for baseline (Figure 1). However, the FCR group had slightly higher scores for both the Utility Index from 24 months and EQ‐VAS from 30 months. Similarly, in the EORTC‐QLQ‐C30 functioning scales, the IR group and FCR group did not have markedly different trajectories of quality of life. However, in the physical, role and social functioning scales and GHS/QoL scale, participants in the FCR group had lower scores at the end of treatment timepoint that subsequently recovered to be similar to the IR group by 12–18 months (Figure 2A). In the EORTC‐QLQ‐C30 symptom scales, the IR and FCR groups did not have markedly different trajectories for the majority of scales (Figure 2B). Overall, fatigue, pain and insomnia had the highest severity scores when considering all the EORTC‐QLQ‐C30 symptom scales. However, in the fatigue subscale, participants in the FCR group had higher scores at the end of treatment time point that declined to be similar to that of the IR group by 12 months, with scores continuing to fall up to 36 months (Figure 2B). In the diarrhoea scale, participants in the FCR group had lower scores at the end of treatment time point that remained consistently lower than the IR group out to 48 months (Figure 2B). The pain subscale showed a similar pattern, although differences between the two groups diminished by 48 months (Figure 2B). In the EORTC‐QLQ‐CLL16 Symptom Scales, in the fatigue, infection and social problems scales, participants in the FCR group had higher scores at the end of treatment time point that recovered to be similar to the IR group by 12 months (Figure 2C). In the disease effects subscale, participants in the FCR group had lower scores at the end of treatment time point that became most pronounced at the 24‐month time point and remained consistently lower than the IR group out to 48 months (Figure 2C). Overall, fatigue and future health had the highest severity scores when considering all the EORTC‐QLQ‐CLL16 symptom scales.

**FIGURE 3:**
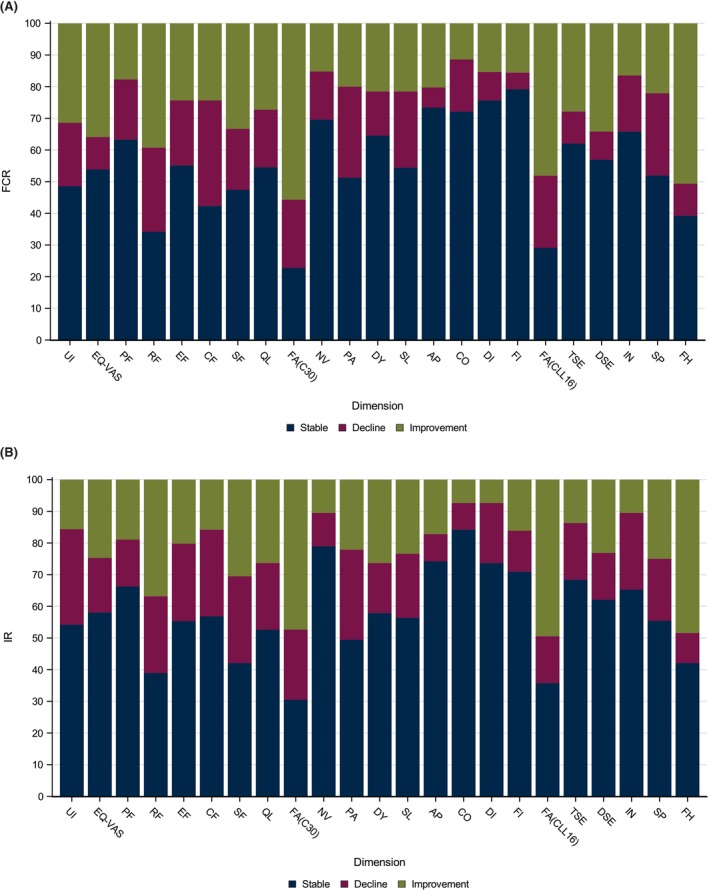
Distribution of changes (declined, improved and remained) in HR‐QoL scales between baseline and 48 m post‐randomisation in (A) FCR and (B) IR. AP, appetite loss; CF, cognitive functioning; CO, constipation; DI, diarhoea; DSE, disease effects; DY, dyspnoea; EF, emotional functioning; EQ‐VAS, EQ5D visual analogue; FA(C30), fatigue; FA(CLL16), fatigue; FH, future health; FI, financial problems; HR‐QoL, health‐related quality of life; IN, infection; NV, nausea/vomiting; PA, pain; PF, physical functioning; QL, global health status/QoL; RF, role functioning; SF, social functioning; SL, insomnia; SP, social problems; TSE, treatment side effects; UI, EQ5D‐3L Utility Index.

In a comparison of baseline and 48‐month time points (Figure 3), the percentage of participants who experienced meaningful improvements was greater in the FCR group for the EQ‐5D Utility Index (IR 15.7%, FCR 31.4%); EQ‐VAS (IR 24.7% FCR, 39.9%); cognitive function (IR 15.8%, FCR 24.4%); fatigue ([C30] IR 47.4%, FCR 55.7%); diarrhoea (IR 7.4%, FCR 15.4%); treatment side effects (IR 13.7%, FCR 27.9%); disease effects (IR 23.2%, FCR 34.2%) and infections (IR 10.5%, FCR 16.5%). The percentage of participants who experienced meaningful deterioration was less in the FCR group for the EQ‐5D Utility Index (IR 30.1%, FCR 20.0%); EQ‐VAS (IR 17.2%, FCR 10.3%); social function (IR 27.3%, FCR 19.2%); diarrhoea (IR 18.9%, FCR 9.0%); financial problems (IR 12.9%, FCR 5.2%); treatment side effects (IR 17.9%, FCR 10.1%); disease effects (IR 14.7%, FCR 8.9%); and infections (IR 24.2%, FCR 17.7%). However, the percentage of participants who experienced a meaningful deterioration was less in the IR group for cognitive function (IR 27.4%, FCR: 33.3%); fatigue ([CLL16] IR 14.7%, FCR 22.8%); constipation (IR 8.4%, FCR 16.5%) and social problems (IR 19.6%, FCR 26.0%).

**FIGURE 4:**
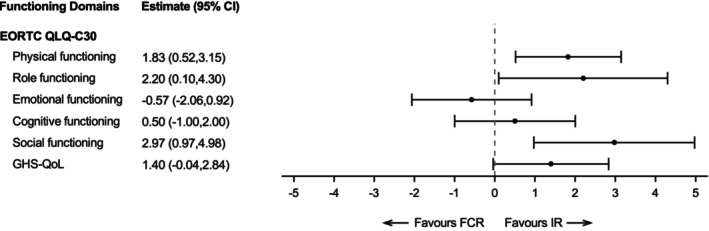
Differences as estimated from repeated measures multi‐level regression models in functioning scales for IR compared with FCR up to month 48. A positive change denotes improvement for the functioning scales of the EORTC QLQ‐C30 (including GHS–QoL) and the EQ‐5D‐3L (Utility Index and VAS). The difference in least‐squares means is in favour of the IR group versus the FCR group when showing positive differences for functioning scales and GHS–QoL. EORTC, European Organisation for Research and Treatment of Cancer; FCR, fludarabine, cyclophosphamide and rituximab; GHS–QoL, global health status–quality of life; IR, ibrutinib and rituximab; QLQ‐C30, Quality of Life C30 Questionnaire.

**FIGURE 5:**
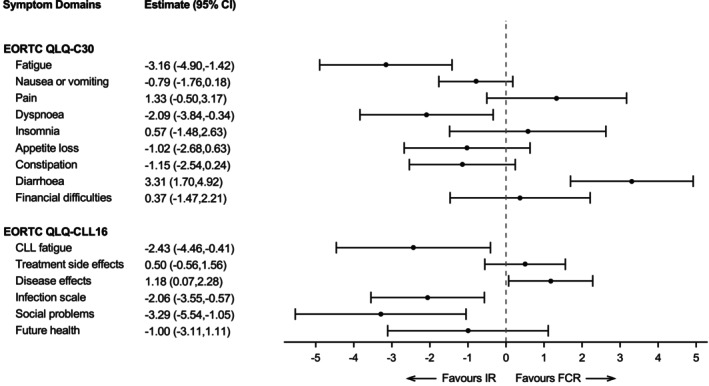
Differences as estimated from repeated measures multilevel regression models in symptom scales for IR compared with FCR up to month 48. A negative change denotes an improvement for the symptom scales of the EORTC QLQ‐C30 and EORTC QLQ‐CLL16. The difference in least‐squares means is in favour of the IR group versus the FCR group when showing negative differences for symptom scales. EORTC, European Organisation for Research and Treatment of Cancer; FCR, fludarabine, cyclophosphamide and rituximab; IR, ibrutinib and rituximab; QLQ‐C30, Quality of Life C30 Questionnaire; QLQ‐CLL16, QLQ CLL Module.

Up to 48 months, the estimated mean differences from repeated measures multilevel modelling were not statistically significant for the EQ‐5D Utility Index or EQ‐VAS (−0.00 [95% CI −0.02, 0.01]; 0.60 [95% CI −0.64, 1.85], Figure S2). In the EORTC‐QLQ‐C30 function scales, physical, role and social function were statistically significant in favour of IR (Figure 4). In the EORTC‐QLQ‐C30 symptom scales, diarrhoea was statistically significant in favour of FCR while fatigue and dyspnoea were in favour of IR (Figure 5). In the EORTC‐QLQ‐CLL16 symptom scales, disease effects were statistically significant in favour of FCR and CLL fatigue, infection and social problems were in favour of IR (Figure 5). However, the observed differences did not exceed the between‐group minimally important difference for improvement for all scales. In sensitivity analyses that excluded participants who completed the baseline questionnaire after randomisation, estimates did not differ markedly (data not shown). Each fitted model included the age stratification factor (≤65 years vs. >65 years) as a covariate, and a small number of models showed age had a small statistically significant effect (*p* < 0.05) on subscales. For emotional functioning, scores were higher in the older age group and for financial problems lower in the older age group generally (Figure S3). However, diarrhoea scores were slightly higher in the younger group in the IR group than in the FCR group and in the older age group (Figure S4).

## DISCUSSION

The FLAIR trial is the largest study comparing ibrutinib‐based therapy (IR) with FCR chemoimmunotherapy, demonstrating superior PFS for IR,^9^ consistent with earlier findings.^22^ Our HR‐QoL data show moderate impairments in physical, role and social function during FCR treatment, likely due to side effects and lifestyle restrictions. Fatigue was worse with FCR, possibly due to cytopenia and appetite loss, while diarrhoea was more common with IR. Social problems were less frequent with IR, reflecting its toxicity profile. In descriptive analysis, contrasting baseline with 48 months post randomisation, there was a higher proportion of meaningful improvements, and a lower proportion of meaningful deterioration, in the FCR group. Despite these differences, HR‐QoL at estimated from multilevel repeated measures models 4 years post‐randomisation among progression‐free survivors was comparable between groups, suggesting that continuous IR is not detrimental to long‐term HR‐QoL.

Compared to age‐matched UK reference populations, FLAIR participants had similar EQ5D‐3L Utility Index scores but lower EQ‐VAS. Role and social function were worse at baseline, possibly due to disease burden, while higher physical function and global health scores may reflect trial eligibility criteria excluding unfit individuals.

Longitudinal HR‐QoL with IR has been underreported. ECOG1912 showed improvements in FACT‐Leu TOI scores with both IR and FCR, with no significant differences between groups.^23^ FLAIR similarly showed moderate improvements in GHS/QoL over time. Physical function declined with FCR but recovered by 12 months, aligning with ECOG1912 findings. Role and social function were more impaired with FCR at end of treatment, but differences diminished over time. Emotional and cognitive functioning showed no significant differences.

The ARCTIC trial^24^, ^25^ also reported longitudinal HR‐QoL improvements with fludarabine‐based regimens. Baseline characteristics and QoL trajectories were similar to FLAIR, suggesting consistency across UK trials over the past decade. This may reflect the younger, fitter trial populations or limitations in current QoL instruments to detect subtle differences in CLL experiences.

Studies of novel agents have shown QoL benefits,^26^ though in a less fit population with reduced intensity chemoimmunotherapy or in a relapsed and refractory population with monotherapy.^27^ In FLAIR, the high baseline QoL may have masked small improvements with FCR. The validated instruments used—EORTC QLQ‐C30, QLQ‐CLL16 and EQ‐5D‐3L—enable cross‐trial comparisons but were developed in the chemoimmunotherapy era. The updated QLQ‐CLL17 includes symptoms relevant to newer therapies like ibrutinib^28^, ^29^ and may have detected more differences. Future trials should consider combining core questionnaires with relevant items from the EORTC library.^30^

In FLAIR, Black participants comprised only 1% of the HR‐QoL cohort, below UK population levels, limiting external validity. Improving recruitment and outcome reporting for ethnic minorities should be prioritised in future CLL trials.

FLAIR collected HR‐QoL data up to disease progression, limiting insights into the impact of relapse. Future trials should extend HR‐QoL assessments beyond first progression.

In conclusion, IR is associated with minimal HR‐QoL differences at 4 years compared to FCR. Such findings may seem counterintuitive given that other QoL studies of novel agents deployed for CLL treatment have demonstrated a favourable impact on QoL when compared to chemo‐immunotherapy.^31^ However, this study was undertaken in a relatively young‐fit patient group who are known to have excellent responses to FCR. This FCR‐treated group may be less likely to have chemotherapy‐related complications than the elderly co‐morbid. UK outcomes for FCR‐treated CLL patients are excellent; however, over long‐time periods, it is likely that continual disease relapses and the significant risk of second malignancies will negatively impact QoL.^25^ IR is an effective and well‐tolerated continuous therapy, in contrast to the time‐limited nature of FCR. The improvements in PFS with this therapy are possibly offset by the known side‐effect profile of ibrutinib (cardiovascular, gastrointestinal and systemic symptoms). Real‐world data of ibrutinib confirm the efficacy of this agent but demonstrates a continual rate to treatment discontinuation due to side effects.^32^

Our results reflect the interplay between time‐limited FCR in a young‐fit patient group and continuous ibrutinib treatment, which, while efficacious, has a burden of toxicity. Newer BTKis may have a more positive impact on QOL, as may time‐limited combinations of novel agents delivered in a personalised approach with MRD monitoring.

## AUTHOR CONTRIBUTIONS

Peter Hillmen and Talha Munir were chief investigators. Peter Hillmen, Dena R. Howard, Anna Hockaday, Julia M. Brown and Talha Munir designed the trial and developed the protocol. E. Faye Samy, Sean Girvan and David A. Cairns developed and carried out the statistical analysis plan. Peter Hillmen, Adrian Bloor, David J. Allsup, Piers E. M. Patten and Talha Munir participated in the recruitment of participants. Andrew Rawstron coordinated the central laboratory investigations. Natasha Greatorex, Sharon Jackson and Anna Hockaday coordinated the data collection and regulatory and governance requirements. Phoebe Templeton, Michael Tupper, David Phillips, Sonam Yaqub, Diana Mortimer and David Stones entered, verified and managed the data collected. David J. Allsup, David A. Cairns, E. Faye Samy, Lelia Duley, Talha Munir and Peter Hillmen interpreted the data. David J. Allsup, David A. Cairns, E. Faye Samy, Lelia Duley, Talha Munir and Peter Hillmen developed the first drafts of the manuscript. E. Faye Samy and David A. Cairns have accessed and verified all the data in the study. All authors had access to all the data reported in the study. All authors contributed to the review and amendments of the manuscript for important intellectual content and approved this final version for submission.

## FUNDING INFORMATION

Primary financial support was from Cancer Research UK (C18027/A15790) to PH. Unrestricted educational grants to PH from Janssen, Pharmacyclics and AbbVie supported trial coordination and laboratory studies. Study drug (ibrutinib) was provided by Janssen. This work was also supported by Core Clinical Trials Unit Infrastructure to DAC, JMB and AH from Cancer Research UK (C7852/A25447).

## CONFLICT OF INTEREST STATEMENT

DJA reports receipt of part of his salary from the National Institute for Health and Care Research and Medical Research Council. Research support from Gilead and Roche; consulting fees from Abbvie; support to attend meetings from CSL Behring BeoOne Medicines, Gilead and Sobi; and membership of board of directors of the Daisy charity. DAC reports unrestricted educational grants to his institution from Janssen, Pharmacyclics and AbbVie; payment to institution for educational lectures from Janssen; participation on a data safety monitoring board for an academically led investigator‐initiated CLL study and personal payment for meeting attendance and report preparation from University Hospital Cologne. AB reports speaker fees from Janssen, Roche and AbbVie and support for conference attendance from Roche and AbbVie. DRH is employed by Roche and holds stock or stock options from Roche. AH reports unrestricted educational grants to her institution from Janssen, Pharmacyclics and AbbVie and receipt of a speaker fee from AbbVie. JMB reports unrestricted educational grants to her institution from Janssen, Pharmacyclics and AbbVie. PEMP reports grants from Roche and Gilead; payment or honoraria for presentations from AbbVie, AstraZeneca, BeiGene, Gilead and Janssen; support for attending meetings or travel from AbbVie; and participation on a data safety monitoring board or advisory board for AbbVie, BeiGene and Novartis. AR reports grants to his institution from AbbVie, Janssen, Pharmacyclics and Roche; consulting fees from BeiGene and Pharmacyclics paid to a company of which AR is a director; payment or honoraria for presentations from AbbVie, Beckman Coulter, BD Biosciences, BeiGene and Janssen paid to a company of which AR is a director; support for attending meetings or travel from Janssen; participation on a data safety monitoring board or advisory board from AbbVie and Janssen; and receipt of equipment from Beckman Coulter. PH reports funding for the study and provision of investigational medicinal products from Janssen and AbbVie; personal consulting fees from Janssen, AbbVie and AstraZeneca; personal speaker fees from Janssen, AbbVie, AstraZeneca and BeiGene; institutional support of clinical trials from Janssen, AbbVie, Gilead Sciences and F Hoffman‐La Roche. TM reports payment for lectures and presentations from Janssen, AbbVie and AstraZeneca; support for attending conferences from Janssen, AbbVie and AstraZeneca; and participation on advisory boards for Janssen, AbbVie, AstraZeneca, Lilly, BeiGene and Morphosys. All other authors declare no competing interests.

## Supporting information


Appendix S1.

## Data Availability

De‐identified participant data will be made available when all trial primary and secondary end‐points have been met. Any requests for trial data and supporting material (data dictionary, protocol and statistical analysis plan) will be reviewed by the trial management group in the first instance. Only requests that have a methodologically sound proposal and whose proposed use of the data has been approved by the independent trial steering committee will be considered. Proposals should be directed to the corresponding author in the first instance; to gain access, data requestors will need to sign a data access agreement.
