# Supplementary material for: Patient‐reported health‐related quality of life in previously untreated chronic lymphocytic leukaemia: Results from the randomised phase 3 FLAIR trial comparing ibrutinib–rituximab versus fludarabine–cyclophosphamide–rituximab
Source: Br J Haematol. 2026 Mar 30;208(5):1596–607. doi: 10.1111/bjh.70416 (PMC13176521; doi:10.1111/bjh.70416)
Supplement: Supplementary file 1 — Appendix S1. [file BJH-208-1596-s001.docx]

**Supplementary Appendix**

**Patient-reported health-related quality of life in previously untreated chronic lymphocytic leukaemia: Results from the randomised phase 3 FLAIR trial comparing ibrutinib–rituximab versus fludarabine–cyclophosphamide–rituximab**

David J Allsup et al.

**Contents**

[Supplementary Methods 2](#_Toc220415265)

[Supplementary Tables 4](#_Toc220415266)

[Table S1: FLAIR Study Sites and Principal Investigators at sites contributing to FLAIR PRO ITT population as indicated by returned baseline questionnaires 4](#_Toc220415267)

[Table S2: Baseline characteristic in the PRO intention-to-treat (ITT) and full ITT populations 8](#_Toc220415268)

[Table S3: Missing forms and scales/items - EQ-5D 10](#_Toc220415269)

[Table S4: Missing forms and scales/items - QLQ C30 11](#_Toc220415270)

[Table S5: Missing forms and scales/items - QLQ CLL16 13](#_Toc220415271)

[Table S6: Summary of participants who completed (any of) the baselines questionnaires after the date of randomisation 14](#_Toc220415272)

[Supplementary Figures 15](#_Toc220415273)

[Figure S1: Summary of compliance to the baseline QoL questionnaires 15](#_Toc220415274)

[Figure S2: Differences as estimated from repeated measures multi-level regression models in functioning scales for IR compared with FCR up to month 48 16](#_Toc220415275)

[Figure S3: Mean scores adjusted for baseline in participants aged less than or equal to 65 years for (a) EQ-5D Utility Index and VAS, (b) EORTC QLQ C30 Functioning Scales, (c) EORTC QLQ C30 Symptom Scales and (d) EORTC QLQ CLL16 Symptom Scales 17](#_Toc220415276)

[Figure S4: Mean scores adjusted for baseline in participants aged greater than 65 years for (a) EQ-5D Utility Index and VAS, (b) EORTC QLQ C30 Functioning Scales, (c) EORTC QLQ C30 Symptom Scales and (d) EORTC QLQ CLL16 Symptom Scales 20](#_Toc220415277)

# Supplementary Methods

**Study design and participants**

There were three major amendments to the standard risk pathway trial design which involved the addition or removal of possible allocated treatment groups and an amendment to add a high-risk pathway (Howard, et al 2021). Primary endpoint results have previously been reported (Hillmen, et al 2023, Munir, et al 2024).

Inclusion criteria for FLAIR were previously untreated patients with CLL/SLL, who were considered fit to receive FCR, were between 18 and 75 years of age with a World Health Organisation (WHO) performance status of two or less and a disease status which required treatment in line with International Workshop on CLL (IWCLL) criteria. Patients with progressive Stage A CLL were eligible for treatment within FLAIR (Hallek*, et al* 2008). In the fourteen days prior to randomisation alanine aminotransferase or aspartate aminotransferase had to be £3x upper limit of normal (ULN) and total bilirubin had to be ≤1.5x ULN (unless bilirubin rise was due to Gilbert’s syndrome or of non-hepatic origin) to be eligible. Key exclusion criteria were Richter’s transformation, Central Nervous System (CNS) involvement, >20% deletion 17p detected on Fluorescence In Situ Hybridization (FISH), symptomatic cardiac disease, and unwillingness to use pregnancy prevention (if indicated). Symptomatic cardiac failure, unstable angina not controlled by current therapy, respiratory impairment and other severe, concurrent diseases or mental disorders that could interfere with ability to participate were also exclusion criteria.

This study was approved by the national ethics review board (National Research Ethics Service, London, UK), institutional review boards at the participating hospitals and the competent regulatory authority (Medicines and Healthcare Products Regulatory Agency, London, UK). FLAIR was conducted in accord with the Declaration of Helsinki and the principles of Good Clinical Practice as espoused in the Medicines for Human Use (Clinical Trials) Regulations.

**Randomisation and masking**

Randomisations were performed at the Clinical Trials Research Unit in Leeds by authorised members of staff with a centralised automated telephone system in accord with a validated minimisation algorithm. Because of the nature of the interventions, the study was open-label, and the allocated treatment was not masked from study investigators or participants. The funders remained masked to treatment results until data cut-off.

**Procedures**

FCR was delivered every twenty-eight days for a total of six cycles in the absence of disease progression or toxicity requiring cessation. Fludarabine and cyclophosphamide were administered orally at doses of 24 and 150 mg/m2/day, respectively, for the first five days of each cycle. Rituximab was administered intravenously at 375 mg/m2 on day 1 of cycle 1 and 500 mg/m2 on day 1 in cycles 2–6. Ibrutinib was administered orally at 420mg/day with rituximab schedule like FCR regimen and then ibrutinib was delivered for either: six years until the MRD stopping rules were reached, toxicity requiring treatment cessation occurred or disease progression was noted, whichever scenario was encountered earliest. Dose reductions and delays were permitted for toxicity and impaired renal function. Prophylaxis with granulocyte colony-stimulating factor was permitted for therapy-induced neutropenia.

Questionnaires were completed in clinic (or were posted to participants) at the following timepoints: prior to randomisation; at the end of treatment with rituximab (FCR/IR); and then every six months from twelve months after randomisation until seven years post-randomisation, or until treatment for progressive disease, whichever occurred earliest. Participants were given an envelope to seal their questionnaire in before returning to their clinical team to preserve confidentiality of results. Participants were given an envelope to seal their questionnaire in before returning to their clinical team to preserve confidentiality of results.

**Statistical Analysis**

The trial was designed to show a 1.5-year increase in median PFS in the IR group (expected median duration of 6 years) compared with the FCR group (expected median duration of 4.5 years, hazard ratio [HR] 0.75) when a total of 379 PFS events had been observed. This calculation assumed that the time-to-event was exponentially distributed and that recruitment would last four years with a further four years of follow-up (Lakatos and Lan 1992). It was based on a two-sided 5% significance level with 80% power. A minimum recruitment target of 748 participants randomly assigned (1:1) to IR or FCR was specified, allowing for a 5% dropout. These assumptions and estimated outcomes with FCR were based upon the results from the CLL8 trial (Molica 2011). There were no sample size or power calculations undertaken for the secondary endpoint of HR-QoL. The EQ-5D-3L consists of five dimensions, each with three levels of severity. These parameters are combined via an algorithm to create a utility index (Dolan 1995). The Visual Analogue Scale (EQ-VAS) measured a participant’s perception of their health ‘today’ on a range from 0 to 100. The QLQ-C30 assesses cancer patients’ functioning, symptoms and overall quality of life. Comprised of a total of thirty questions, an algorithm is used to generate five functional scales, three symptom scales, six single item scales and a global health status (GHS) and QoL scale which ranges from 0 to 100. The QLQ-CLL16 considers specifically the HR-QoL of persons with CLL with sixteen symptom-related questions which are combined to form four multi-item scales and two single item scales.

The time points (ranging from baseline to forty-eight months) were categorical and defined by the questionnaire time label rather than time calculated from randomisation. The nature of missing data was determined to be missing at random (MAR) by considering data patterns.

In the multi-level repeated measures model, three models were considered: (1) a random intercept model which included the fixed effects such as allocated treatment, time as a continuous variable (months), age group, gender, Binet stage and baseline scale; (2) the addition of time as a random effect, retaining the same fixed effects as in (1); (3) the introduction of an interaction term between treatment and time as a fixed effect in addition to the terms described in (2). The addition of a quadratic time term to the model was also investigated.

These models considered four different variance-covariance structures: compound symmetry, unstructured, autoregressive and autoregressive heterogeneous variances. The Akaike Information Criterion and Bayesian Information Criterion were used to determine the best model fit for each scale. The time of questionnaire completion was calculated from the date of randomisation to the date of completion. Any questionnaires with a completion date later than forty-eight months from randomisation were excluded from the analysis.

**Role of the funding source**

The funders of the study had no role in study design, data collection, data analysis, data interpretation, or writing of the report. The corresponding author had full access to all the data in the study and had final responsibility for the decision to submit for publication.

**References**

Dolan, P., et al. (1995) A social tariff for EuroQol: results from a UK general population survey., University of York, Centre for Health Economics York.

Hallek, M., Cheson, B.D., Catovsky, D., Caligaris-Cappio, F., Dighiero, G., Dohner, H., Hillmen, P., Keating, M.J., Montserrat, E., Rai, K.R., Kipps, T.J. & International Workshop on Chronic Lymphocytic, L. (2008) Guidelines for the diagnosis and treatment of chronic lymphocytic leukemia: a report from the International Workshop on Chronic Lymphocytic Leukemia updating the National Cancer Institute-Working Group 1996 guidelines. *Blood,* **111,** 5446-5456.

Hillmen, P., Pitchford, A., Bloor, A., Broom, A., Young, M., Kennedy, B., Walewska, R., Furtado, M., Preston, G., Neilson, J.R., Pemberton, N., Sidra, G., Morley, N., Cwynarski, K., Schuh, A., Forconi, F., Elmusharaf, N., Paneesha, S., Fox, C.P., Howard, D.R., Hockaday, A., Brown, J.M., Cairns, D.A., Jackson, S., Greatorex, N., Webster, N., Shingles, J., Dalal, S., Patten, P.E.M., Allsup, D., Rawstron, A. & Munir, T. (2023) Ibrutinib and rituximab versus fludarabine, cyclophosphamide, and rituximab for patients with previously untreated chronic lymphocytic leukaemia (FLAIR): interim analysis of a multicentre, open-label, randomised, phase 3 trial. *Lancet Oncol,* **24,** 535-552.

Howard, D.R., Hockaday, A., Brown, J.M., Gregory, W.M., Todd, S., Munir, T., Oughton, J.B., Dimbleby, C. & Hillmen, P. (2021) A platform trial in practice: adding a new experimental research arm to the ongoing confirmatory FLAIR trial in chronic lymphocytic leukaemia. *Trials,* **22,** 38.

Lakatos, E. & Lan, K.K. (1992) A comparison of sample size methods for the logrank statistic. *Stat Med,* **11,** 179-191.

Molica, S. (2011) Progress in the treatment of chronic lymphocytic leukemia: results of the German CLL8 trial. *Expert Rev Anticancer Ther,* **11,** 1333-1340.

Munir, T., Cairns, D.A., Bloor, A., Allsup, D., Cwynarski, K., Pettitt, A., Paneesha, S., Fox, C.P., Eyre, T.A., Forconi, F., Elmusharaf, N., Kennedy, B., Gribben, J., Pemberton, N., Sheehy, O., Preston, G., Schuh, A., Walewska, R., Duley, L., Howard, D., Hockaday, A., Jackson, S., Greatorex, N., Girvan, S., Bell, S., Brown, J.M., Webster, N., Dalal, S., de Tute, R., Rawstron, A., Patten, P.E.M., Hillmen, P. & National Cancer Research Institute Chronic Lymphocytic Leukemia, S. (2024) Chronic Lymphocytic Leukemia Therapy Guided by Measurable Residual Disease. *N Engl J Med,* **390,** 326-337.

# Supplementary Tables

## Table S1: FLAIR Study Sites and Principal Investigators at sites contributing to FLAIR PRO ITT population as indicated by returned baseline questionnaires

| **Centre name** | **Principal Investigators** | **Number of baseline questionnaires** |
| --- | --- | --- |
| Kings College Hospital, London | Dr Piers Patten | 27 |
| University Hospital of Wales, Cardiff | Dr Chris Fegan, Dr Nagah Elmusharaf, Dr Simona Gatto | 23 |
| Southampton General Hospital | Dr Francesco Forconi | 22 |
| The Christie Hospital, Manchester | Professor Adrian Bloor | 22 |
| Nottingham University Hospital | Dr Christopher Fox | 21 |
| Royal Hallamshire Hospital, Sheffield | Dr Nick Morley | 21 |
| University College London Hospital | Dr Kate Cwynarksi, Dr Satyen Gohil | 21 |
| Churchill Hospital, Oxford | Dr Anna Schuh, Dr Toby Eyre | 20 |
| Castle Hill Hospital, Hull | Dr David Allsup | 18 |
| Russell's Hall Hospital, Dudley | Dr Jeff Neilson | 16 |
| Worcestershire Acute Hospitals NHS Trust | Dr Nicholas Pemberton | 16 |
| St James University Hospital, Leeds | Professor Peter Hillmen, Dr Talha Munir | 15 |
| Aberdeen Royal Infirmary | Dr Caroline Duncan, Dr Gavin Preston | 14 |
| Royal Bournemouth Hospital | Dr Renata Walewska | 14 |
| Royal Cornwall Hospital, Truro | Dr Julie Blundell, Dr Richard Noble, Dr Michelle Furtado | 14 |
| Kent and Canterbury Hospital | Dr Chris Pocock, Dr Moya Young | 13 |
| Birmingham Heartlands Hospital | Dr Shankara Paneesha | 12 |
| Good Hope Hospital, Birmingham | Dr Shankara Paneesha | 12 |
| Leicester Royal Infirmary | Dr Ben Kennedy, Dr Constantinos Balotis | 12 |
| Western General Hospital, Edinburgh | Dr Fiona Scott, Dr Angus Broom | 12 |
| Blackpool Victoria Hospital | Dr Marian Macheta | 11 |
| University Hospitals of North Midlands NHS Trust (Royal Stoke) | Dr Andrew Stewart, Dr Paul Ferguson, Dr Neil Phillips | 11 |
| Buckinghamshire Healthcare NHS Trust | Dr Jonathan Pattinson, Dr Helen Eagleton | 9 |
| Clatterbridge Cancer Centre, Liverpool | Professor Andrew Pettitt | 9 |
| Colchester General Hospital | Dr Michael Hamblin, Dr Gavin Preston, Dr Mahalakshmi Mohan | 9 |
| Milton Keynes Hospital | Dr Moez Dungarwalla | 9 |
| Queen Elizabeth Hospital, Birmingham | Dr Paul Moss, Dr Jim Murray, Professor Guy Pratt | 9 |
| Royal Surrey County Hospital, Guildford | Dr Johannes De Vos , Dr Elisabeth Grey-Davies | 9 |
| Worthing Hospital | Dr Santosh Narat | 9 |
| Epsom and St Helier Hospital | Dr Jane Mercieca, Dr Lydia Jones, Dr Corrine De Lord, Dr Farheen Mir, Dr Simon Stern | 8 |
| Manchester Royal Infirmary | Dr Sarah Burns | 8 |
| Salisbury District Hospital | Dr Jonathan Cullis, Dr James Milnthorpe | 8 |
| Southmead Hospital, Bristol | Dr Sophie Otton | 8 |
| St Bartholomew's Hospital, London | Professor John Gribben | 8 |
| Bradford Royal Infirmary | Dr Adrian Williams, Dr Anshu Garg, Dr Abida Naeem | 7 |
| Royal Derby Hospital | Dr Jo Addada, Dr Meghna Ruparelia | 7 |
| Royal Devon Exeter Hospital | Dr Anthony Todd | 7 |
| Addenbrooke's Hospital, Cambridge | Dr George Follows | 6 |
| Beatson Oncology Centre, Glasgow | Dr Alison McCaig, Dr Mark Rafferty | 6 |
| Belfast City Hospital | Dr Oonagh Sheehy | 6 |
| Grantham and District General Hospital | Dr Gamal Sidra | 6 |
| Lincoln County Hospital | Dr Gamal Sidra | 6 |
| Musgrove Park Hospital, Taunton | Dr Belinda Austen | 6 |
| Queen Elizabeth Hospital, Gateshead | Dr Scott Marshall | 6 |
| Royal Marsden Hospital, London | Dr Claire Dearden, Dr Sunil Iyengar | 6 |
| Singleton Hospital, Swansea | Dr Unmesh Mohite | 6 |
| Torbay District General Hospital, Torquay | Dr Deborah Turner | 6 |
| Ysbyty Gwynedd | Dr Earnest Heartin, Dr Sally Evans | 6 |
| Bristol Haematology and Oncology Centre | Dr Lisa Lowry, Dr Rachel Protheroe, Dr Nikesh Chavda | 5 |
| Doncaster Royal Infirmary | Dr Youssef Sorour, Dr Joe Joseph, Dr Atchamamba Bobbili, Dr Sophie Todd, Dr Tobore Gbemre | 5 |
| Glan Clwyd Hospital | Dr Earnest Heartin | 5 |
| Pilgrim Hospital, Boston | Dr Gamal Sidra | 5 |
| Raigmore Hospital | Dr Chris Lush, Dr Catherine Ogilvie, Dr Caroline Duncan | 5 |
| Rotherham General Hospital | Dr Richard Went, Dr Kathryn Goddard | 5 |
| York Hospital | Dr Annika Whittle | 5 |
| George Elliot Hospital, Nuneaton | Dr Jagdesh Gandla, Dr Jhansi Muddana | 4 |
| Harrogate District Hospital | Dr Tharani Balasubramaniam, Dr Claire Hall | 4 |
| NHS Lanarkshire | Dr Lindsay Mitchell | 4 |
| Northampton General Hospital | Dr Sajjan Mittal | 4 |
| Pennine Acute Hospitals NHS Trust | Dr Antonina Zhelyazkova | 4 |
| Salford Royal Hospital | Dr Rowena Thomas-Dewing, Dr Sonya Ravenscroft | 4 |
| Sandwell General Hospital, West Bromwich | Dr Yasmin Hasan | 4 |
| Scunthorpe General Hospital | Dr Sanjeev Jalihal, Dr Afzal Ponnambath | 4 |
| St Georges Hospital, London | Dr Fanella Willis | 4 |
| St Richards Hospital, Worthing | Dr Santosh Narat | 4 |
| Wrexham Maelor Hospital | Dr David Watson, Dr Lally Desoysa | 4 |
| Basildon Hospital | Dr Parag Jasani, Dr Sadhakaran Makkuni | 3 |
| Calderdale Royal Hospital, Huddersfield | Dr Kate Rothwell | 3 |
| Craigavon Area Hospital | Dr Abdulhakim Eswedi, Dr Kathryn Boyd, Dr Christina Bradford | 3 |
| Croydon University Hospital | Dr Edward Truelove, Dr Betty Cheung | 3 |
| Glangwili General Hospital, Carmarthen | Dr Peter Cumber, Dr Saran Nicholas | 3 |
| Gloucestershire Royal Hospital | Dr Rebecca Frewin, Dr Richard Lush, Dr Rory McCulloch | 3 |
| Ipswich Hospital | Dr Isobel Chalmers | 3 |
| New Victoria Hospital Glasgow | Dr Alison McCaig | 3 |
| Queen Alexandra Hospital Portsmouth | Dr Edward Belsham | 3 |
| Queen Margaret Hospital, Dunfermline | Dr Kerri Davidson | 3 |
| Royal Albert and Edward Infirmary and Thomas Linacre Outpatient Centre, Wigan | Dr Hitesh Patel, Dr Chris Gregory | 3 |
| Scarborough Hospital | Dr Laura Munro | 3 |
| Surrey and Sussex Healthcare NHS Trust | Dr Pawel Kaczmarek | 3 |
| University Hospital Aintree | Dr Jeffrey Smith, Dr Vikram Singh | 3 |
| Altnagelvin Hospital | Dr Patrick Elder | 2 |
| Hampshire Hospitals NHS Foundation Trust | Dr Sylwia Simpson, Dr Jenny Arnold | 2 |
| Huddersfield Royal Infirmary | Dr Kate Rothwell | 2 |
| Kings Mill Hospital, Mansfield | Dr Steve Jones | 2 |
| Princess Royal University Hospital, Orpington | Dr Piers Patten | 2 |
| Queens Hospital, Romford | Dr Paul Greaves | 2 |
| Royal Alexandra Hospital, Paisley | Dr Alison McCaig, Dr Alison Sefcick | 2 |
| South Tees NHS Foundation Trust | Dr Rajesh Mamadigi, Dr Jamie Maddox | 2 |
| UHSM/Wythenshawe | Dr Simon Watt | 2 |
| University Hospital Coventry | Dr Anand Lokare, Dr Sarah Nicolle | 2 |
| Royal Hampshire County Hospital, Winchester | Dr Sylwia Simpson, Dr Jenny Arnold | 1 |
| Borders General Hospital, Melrose | Dr Fiona Scott, Dr Jennifer Buxton, Dr Srinivasa Dasari, Dr Rosemary Jones | 1 |
| Cheltenham General Hospital | Dr Rebecca Frewin, Dr Richard Lush, Dr Rory McCulloch | 1 |
| Diana Princess of Wales Hospital, Grimsby | Dr Sanjeev Jalihal, Dr Afzal Ponnambath | 1 |
| Great Western Hospital, Swindon | Dr Norbert Blesing, Dr Alex Sternberg | 1 |
| James Paget Hospital, Great Yarmouth | Dr Shalal Sadullah, Dr Roberto Consuegra | 1 |
| Nevill Hall Hospital, Abergavenny | Dr Nilima Parry-Jones | 1 |
| Victoria Hospital, Kirkcaldy | Dr Kerri Davidson | 1 |

## Table S2: Baseline characteristic in the PRO intention-to-treat (ITT) and full ITT populations

Abbreviations: CLL, Chronic lymphocytic leukaemia; WHO, World Health Organisation.

|  | **QoL population (n=651)** | **ITT population (n=771)** |
| --- | --- | --- |
| **Gender** |  |  |
| Male | 477 (73.3%) | 565 (73.3%) |
| Female | 174 (26.7%) | 206 (26.7%) |
|  |  |  |
| **Age** |  |  |
| Median (range) | 63.1 (27.0, 75.7) | 62.8 (27.0, 75.7) |
| =< 65 years | 428 (65.7%) | 514 (66.7%) |
| > 65 years | 223 (34.3%) | 257 (33.3%) |
|  |  |  |
| **Binet Stage** |  |  |
| Progressive A or B | 370 (56.8%) | 435 (56.4%) |
| C | 281 (43.2%) | 336 (43.6%) |
|  |  |  |
| **Ethnicity** |  |  |
| White | 620 (95.2%) | 729 (94.6%) |
| Mixed - White and Black Caribbean | 0 (0.0%) | 0 (0.0%) |
| Mixed - White and Black African | 0 (0.0%) | 0 (0.0%) |
| Mixed - White and Asian | 1 (0.2%) | 1 (0.1%) |
| Other mixed background | 1 (0.2%) | 1 (0.1%) |
| Asian - Indian | 2 (0.3%) | 4 (0.5%) |
| Asian - Pakistani | 1 (0.2%) | 1 (0.1%) |
| Asian - Bangladeshi | 0 (0.0%) | 0 (0.0%) |
| Other Asian background | 0 (0.0%) | 1 (0.1%) |
| Black - Caribbean | 4 (0.6%) | 5 (0.6%) |
| Black - African | 3 (0.5%) | 3 (0.4%) |
| Other Black background | 1 (0.2%) | 1 (0.1%) |
| Chinese | 0 (0.0%) | 0 (0.0%) |
| Other ethnic group | 2 (0.3%) | 4 (0.5%) |
| Not stated | 16 (2.5%) | 21 (2.7%) |
| Other | 0 (0.0%) | 0 (0.0%) |
|  |  |  |
| **Duration of CLL (months)** |  |  |
| Mean (s.d.) | 35.2 (36.5) | 35.2 (36.2) |
| Median (range) | 24.1 (0.00, 219) | 24.1 (0.00, 219) |
| Missing | 60 | 78 |
|  |  |  |
| **WHO performance status** |  |  |
| 0 | 430 (66.1%) | 507 (65.8%) |
| 1 | 203 (31.2%) | 244 (31.6%) |
| 2 | 16 (2.5%) | 18 (2.3%) |
| 3 | 0 (0.0%) | 0 (0.0%) |
| 4 | 0 (0.0%) | 0 (0.0%) |
| Missing | 2 (0.3%) | 2 (0.3%) |
|  |  |  |

## Table S3: Missing forms and scales/items - EQ-5D

| **Timepoint** | **Forms missing n/N(%)** | **Forms received N** | **Utility Index** **n(%)** | **EQ-VAS scale n(%)** |
| --- | --- | --- | --- | --- |
| Baseline | 0/651 (0.0) | 651 | 0 (0.0) | 0 (0.0) |
| End of FCR/R | 108/639 (16.9) | 531 | 19 (3.6) | 17 (3.2) |
| 12M | 112/615 (18.2) | 503 | 23 (4.6) | 11 (2.2) |
| 18M | 107/591 (18.1) | 484 | 19 (3.9) | 7 (1.4) |
| 24M | 95/577 (16.5) | 482 | 39 (8.1) | 8 (1.7) |
| 30M | 124/557 (22.3) | 433 | 29 (6.7) | 9 (2.1) |
| 36M | 132/535 (24.7) | 403 | 34 (8.4) | 8 (2.0) |
| 42M | 154/523 (29.4) | 369 | 37 (10.0) | 2 (0.5) |
| 48M | 147/463 (31.7) | 316 | 41 (13.0) | 4 (1.3) |
| 54M | 161/406 (39.7) | 245 | 21 (8.6) | 4 (1.6) |
| 60M | 123/312 (39.4) | 189 | 22 (11.6) | 0 (0.0) |
| 66M | 110/227 (48.5) | 117 | 6 (5.1) | 1 (0.9) |
| 72M | 78/143 (54.5) | 65 | 4 (6.2) | 1 (1.5) |
| 78M | 52/79 (65.8) | 27 | 0 (0.0) | 0 (0.0) |
| 84M | 14/17 (82.4) | 3 | 0 (0.0) | 0 (0.0) |

Missing forms are considered from those that were expected in patients with an evaluable baseline questionnaire.

The denominator for the scale/item percentage calculations is the number of forms received at the specified time point.

All received forms are considered, regardless of whether or not they were in the specified window.

## Table S4: Missing forms and scales/items - QLQ C30

| **Timepoint** | **Forms missing n/N(%)** | **Forms received N** | **Global health/QoL n(%)** | **Physical n(%)** | **Role n(%)** | **Emotional n(%)** | **Cognitive n(%)** | **Social n(%)** | **Fatigue n(%)** | **Nausea/ Vomiting n(%)** | **Pain n(%)** |
| --- | --- | --- | --- | --- | --- | --- | --- | --- | --- | --- | --- |
| Baseline | 0/651 (0.0) | 651 | 0 (0.0) | 0 (0.0) | 0 (0.0) | 0 (0.0) | 0 (0.0) | 0 (0.0) | 0 (0.0) | 0 (0.0) | 0 (0.0) |
| End of FCR/R | 111/639 (17.4) | 528 | 9 (1.7) | 3 (0.6) | 3 (0.6) | 8 (1.5) | 7 (1.3) | 7 (1.3) | 4 (0.8) | 4 (0.8) | 0 (0.0) |
| 12M | 120/615 (19.5) | 495 | 8 (1.6) | 2 (0.4) | 1 (0.2) | 7 (1.4) | 6 (1.2) | 6 (1.2) | 1 (0.2) | 1 (0.2) | 0 (0.0) |
| 18M | 108/591 (18.3) | 483 | 3 (0.6) | 4 (0.8) | 2 (0.4) | 3 (0.6) | 2 (0.4) | 3 (0.6) | 3 (0.6) | 4 (0.8) | 1 (0.2) |
| 24M | 100/577 (17.3) | 477 | 6 (1.3) | 6 (1.3) | 5 (1.0) | 6 (1.3) | 6 (1.3) | 6 (1.3) | 6 (1.3) | 6 (1.3) | 2 (0.4) |
| 30M | 128/557 (23.0) | 429 | 8 (1.9) | 3 (0.7) | 3 (0.7) | 8 (1.9) | 7 (1.6) | 10 (2.3) | 3 (0.7) | 4 (0.9) | 2 (0.5) |
| 36M | 130/535 (24.3) | 405 | 5 (1.2) | 7 (1.7) | 7 (1.7) | 5 (1.2) | 6 (1.5) | 6 (1.5) | 8 (2.0) | 7 (1.7) | 0 (0.0) |
| 42M | 160/523 (30.6) | 363 | 5 (1.4) | 3 (0.8) | 2 (0.6) | 4 (1.1) | 5 (1.4) | 4 (1.1) | 2 (0.6) | 3 (0.8) | 0 (0.0) |
| 48M | 148/463 (32.0) | 315 | 3 (1.0) | 1 (0.3) | 1 (0.3) | 3 (1.0) | 2 (0.6) | 2 (0.6) | 1 (0.3) | 1 (0.3) | 0 (0.0) |
| 54M | 162/406 (39.9) | 244 | 2 (0.8) | 2 (0.8) | 3 (1.2) | 2 (0.8) | 2 (0.8) | 4 (1.6) | 3 (1.2) | 3 (1.2) | 0 (0.0) |
| 60M | 127/312 (40.7) | 185 | 1 (0.5) | 2 (1.1) | 1 (0.5) | 2 (1.1) | 1 (0.5) | 1 (0.5) | 1 (0.5) | 1 (0.5) | 0 (0.0) |
| 66M | 109/227 (48.0) | 118 | 2 (1.7) | 0 (0.0) | 0 (0.0) | 2 (1.7) | 2 (1.7) | 2 (1.7) | 1 (0.8) | 0 (0.0) | 0 (0.0) |
| 72M | 79/143 (55.2) | 64 | 0 (0.0) | 0 (0.0) | 0 (0.0) | 0 (0.0) | 0 (0.0) | 0 (0.0) | 0 (0.0) | 0 (0.0) | 0 (0.0) |
| 78M | 52/79 (65.8) | 27 | 0 (0.0) | 0 (0.0) | 0 (0.0) | 0 (0.0) | 0 (0.0) | 0 (0.0) | 0 (0.0) | 0 (0.0) | 0 (0.0) |
| 84M | 14/17 (82.4) | 3 | 0 (0.0) | 0 (0.0) | 0 (0.0) | 0 (0.0) | 0 (0.0) | 0 (0.0) | 0 (0.0) | 0 (0.0) | 0 (0.0) |

Missing forms are considered from those that were expected in patients with an evaluable baseline questionnaire.

The denominator for the scale/item percentage calculations is the number of forms received at the specified time point.

All received forms are considered, regardless of whether or not they were in the specified window.

**Table S4: continued: Missing forms and scales/items - QLQ C30**

| **Timepoint** | **Short of breath n(%)** | **Trouble sleeping n(%)** | **Lacked appetite n(%)** | **Constipated n(%)** | **Diarrhoea n(%)** | **Financial Difficulties n(%)** |
| --- | --- | --- | --- | --- | --- | --- |
| Baseline | 0 (0.0) | 0 (0.0) | 0 (0.0) | 0 (0.0) | 0 (0.0) | 0 (0.0) |
| End of FCR/R | 5 (0.9) | 6 (1.1) | 7 (1.3) | 6 (1.1) | 7 (1.3) | 10 (1.9) |
| 12M | 4 (0.8) | 1 (0.2) | 1 (0.2) | 2 (0.4) | 6 (1.2) | 8 (1.6) |
| 18M | 4 (0.8) | 3 (0.6) | 5 (1.0) | 5 (1.0) | 6 (1.2) | 4 (0.8) |
| 24M | 7 (1.5) | 8 (1.7) | 7 (1.5) | 7 (1.5) | 6 (1.3) | 7 (1.5) |
| 30M | 5 (1.2) | 6 (1.4) | 4 (0.9) | 3 (0.7) | 10 (2.3) | 13 (3.0) |
| 36M | 13 (3.2) | 9 (2.2) | 8 (2.0) | 11 (2.7) | 8 (2.0) | 9 (2.2) |
| 42M | 4 (1.1) | 4 (1.1) | 4 (1.1) | 3 (0.8) | 5 (1.4) | 6 (1.7) |
| 48M | 2 (0.6) | 3 (1.0) | 5 (1.6) | 1 (0.3) | 3 (1.0) | 7 (2.2) |
| 54M | 5 (2.0) | 3 (1.2) | 4 (1.6) | 5 (2.0) | 4 (1.6) | 5 (2.0) |
| 60M | 1 (0.5) | 2 (1.1) | 1 (0.5) | 2 (1.1) | 2 (1.1) | 1 (0.5) |
| 66M | 1 (0.8) | 0 (0.0) | 0 (0.0) | 0 (0.0) | 2 (1.7) | 3 (2.5) |
| 72M | 0 (0.0) | 0 (0.0) | 0 (0.0) | 1 (1.6) | 0 (0.0) | 1 (1.6) |
| 78M | 1 (3.7) | 0 (0.0) | 0 (0.0) | 1 (3.7) | 0 (0.0) | 0 (0.0) |
| 84M | 0 (0.0) | 0 (0.0) | 0 (0.0) | 0 (0.0) | 0 (0.0) | 0 (0.0) |

Missing forms are considered from those that were expected in patients with an evaluable baseline questionnaire.

The denominator for the scale/item percentage calculations is the number of forms received at the specified time point.

All received forms are considered, regardless of whether or not they were in the specified window.

## Table S5: Missing forms and scales/items - QLQ CLL16

| **Timepoint** | **Forms missing n/N(%)** | **Forms received N** | **Fatigue n(%)** | **Treatment Side Effects n(%)** | **Disease Effects n(%)** | **Infection Scale n(%)** | **Limited in planning activities n(%)** | **Worried about future health n(%)** |
| --- | --- | --- | --- | --- | --- | --- | --- | --- |
| Baseline | 0/651 (0.0) | 651 | 0 (0.0) | 0 (0.0) | 0 (0.0) | 0 (0.0) | 0 (0.0) | 0 (0.0) |
| End of FCR/R | 116/639 (18.2) | 523 | 0 (0.0) | 0 (0.0) | 0 (0.0) | 2 (0.4) | 3 (0.6) | 3 (0.6) |
| 12M | 125/615 (20.3) | 490 | 2 (0.4) | 0 (0.0) | 1 (0.2) | 1 (0.2) | 4 (0.8) | 4 (0.8) |
| 18M | 108/591 (18.3) | 483 | 1 (0.2) | 1 (0.2) | 0 (0.0) | 1 (0.2) | 6 (1.2) | 7 (1.4) |
| 24M | 103/577 (17.9) | 474 | 1 (0.2) | 1 (0.2) | 1 (0.2) | 1 (0.2) | 6 (1.3) | 2 (0.4) |
| 30M | 133/557 (23.9) | 424 | 1 (0.2) | 2 (0.5) | 1 (0.2) | 0 (0.0) | 7 (1.7) | 2 (0.5) |
| 36M | 134/535 (25.0) | 401 | 0 (0.0) | 1 (0.2) | 0 (0.0) | 0 (0.0) | 5 (1.2) | 4 (1.0) |
| 42M | 165/523 (31.5) | 358 | 1 (0.3) | 1 (0.3) | 0 (0.0) | 1 (0.3) | 5 (1.4) | 3 (0.8) |
| 48M | 150/463 (32.4) | 313 | 0 (0.0) | 0 (0.0) | 0 (0.0) | 0 (0.0) | 6 (1.9) | 2 (0.6) |
| 54M | 164/406 (40.4) | 242 | 0 (0.0) | 0 (0.0) | 1 (0.4) | 0 (0.0) | 7 (2.9) | 2 (0.8) |
| 60M | 127/312 (40.7) | 185 | 1 (0.5) | 0 (0.0) | 1 (0.5) | 1 (0.5) | 4 (2.2) | 1 (0.5) |
| 66M | 111/227 (48.9) | 116 | 0 (0.0) | 0 (0.0) | 0 (0.0) | 0 (0.0) | 2 (1.7) | 0 (0.0) |
| 72M | 79/143 (55.2) | 64 | 0 (0.0) | 0 (0.0) | 0 (0.0) | 0 (0.0) | 0 (0.0) | 1 (1.6) |
| 78M | 52/79 (65.8) | 27 | 0 (0.0) | 0 (0.0) | 0 (0.0) | 0 (0.0) | 0 (0.0) | 0 (0.0) |
| 84M | 14/17 (82.4) | 3 | 0 (0.0) | 0 (0.0) | 0 (0.0) | 0 (0.0) | 0 (0.0) | 0 (0.0) |

Missing forms are considered from those that were expected in patients with an evaluable baseline questionnaire.

The denominator for the scale/item percentage calculations is the number of forms received at the specified time point.

All received forms are considered, regardless of whether or not they were in the specified window.

## Table S6: Summary of participants who completed (any of) the baselines questionnaires after the date of randomisation

|  | **FCR (n=353)** | **IR (n=353)** | **Total (n=706)** |
| --- | --- | --- | --- |
| **Baseline questionnaire completed post-randomisation** |  |  |  |
| Yes | 83 (23.5%) | 72 (20.4%) | 155 (22.0%) |
| No | 270 (76.5%) | 281 (79.6%) | 551 (78.0%) |
| **Baseline questionnaire completed more than 7 days post-randomisation** |  |  |  |
| Yes | 43 (12.2%) | 44 (12.5%) | 87 (12.3%) |
| No | 310 (87.8%) | 309 (87.5%) | 619 (87.7%) |

# Supplementary Figures

## Figure S1: Summary of compliance to the baseline QoL questionnaires


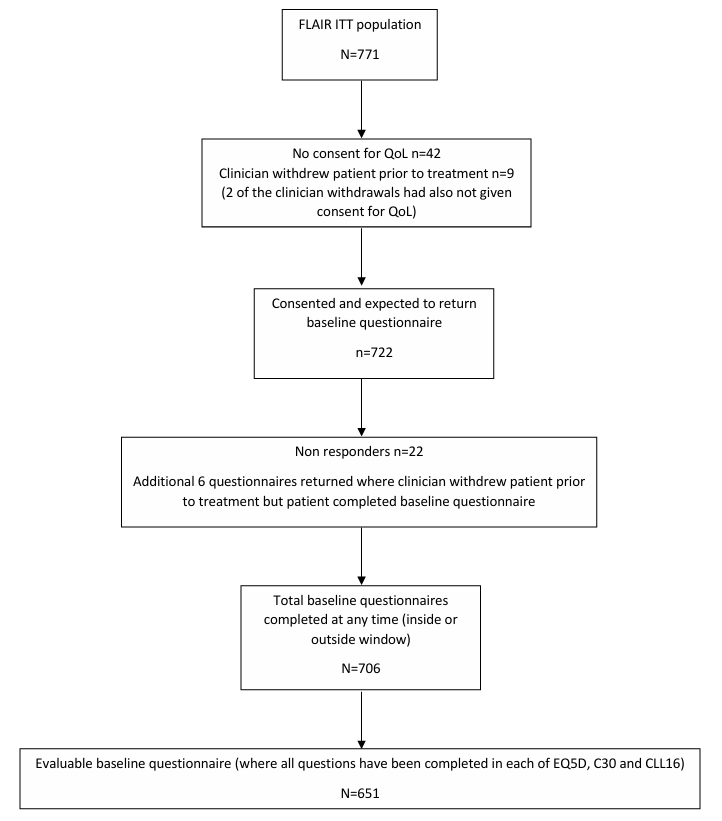


## Figure S2: Differences as estimated from repeated measures multi-level regression models in functioning scales for IR compared with FCR up to month 48


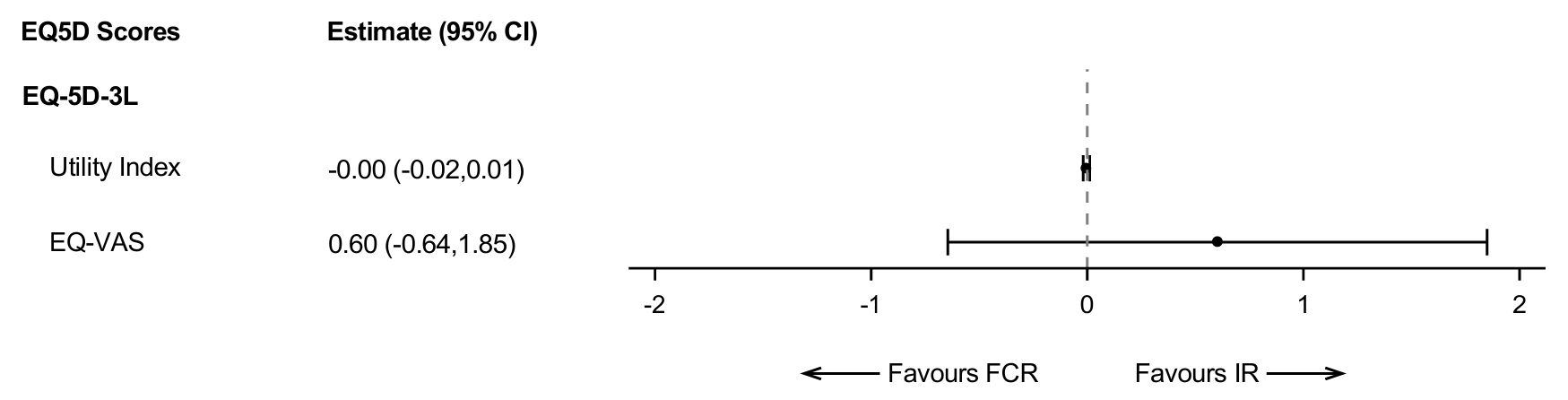


A positive least-squares mean change denotes improvement for the functioning scales of the EORTC QLQ-C30 (including GHS–QoL) and the EQ-5D-3L (Utility Index and VAS). The difference in least-squares means is in favour of the IR group versus the FCR group when showing positive differences for functioning scales and GHS–QoL. EORTC, European Organisation for Research and Treatment of Cancer; QLQ-C30, Quality of Life C30 Questionnaire; GHS–QoL=global health status–quality of life; IR, ibrutinib and rituximab; FCR, fludarabine, cyclophosphamide and rituximab.

## Figure S3: Mean scores adjusted for baseline in participants aged less than or equal to 65 years for (a) EQ-5D Utility Index and VAS, (b) EORTC QLQ C30 Functioning Scales, (c) EORTC QLQ C30 Symptom Scales and (d) EORTC QLQ CLL16 Symptom Scales

**(a)**

**
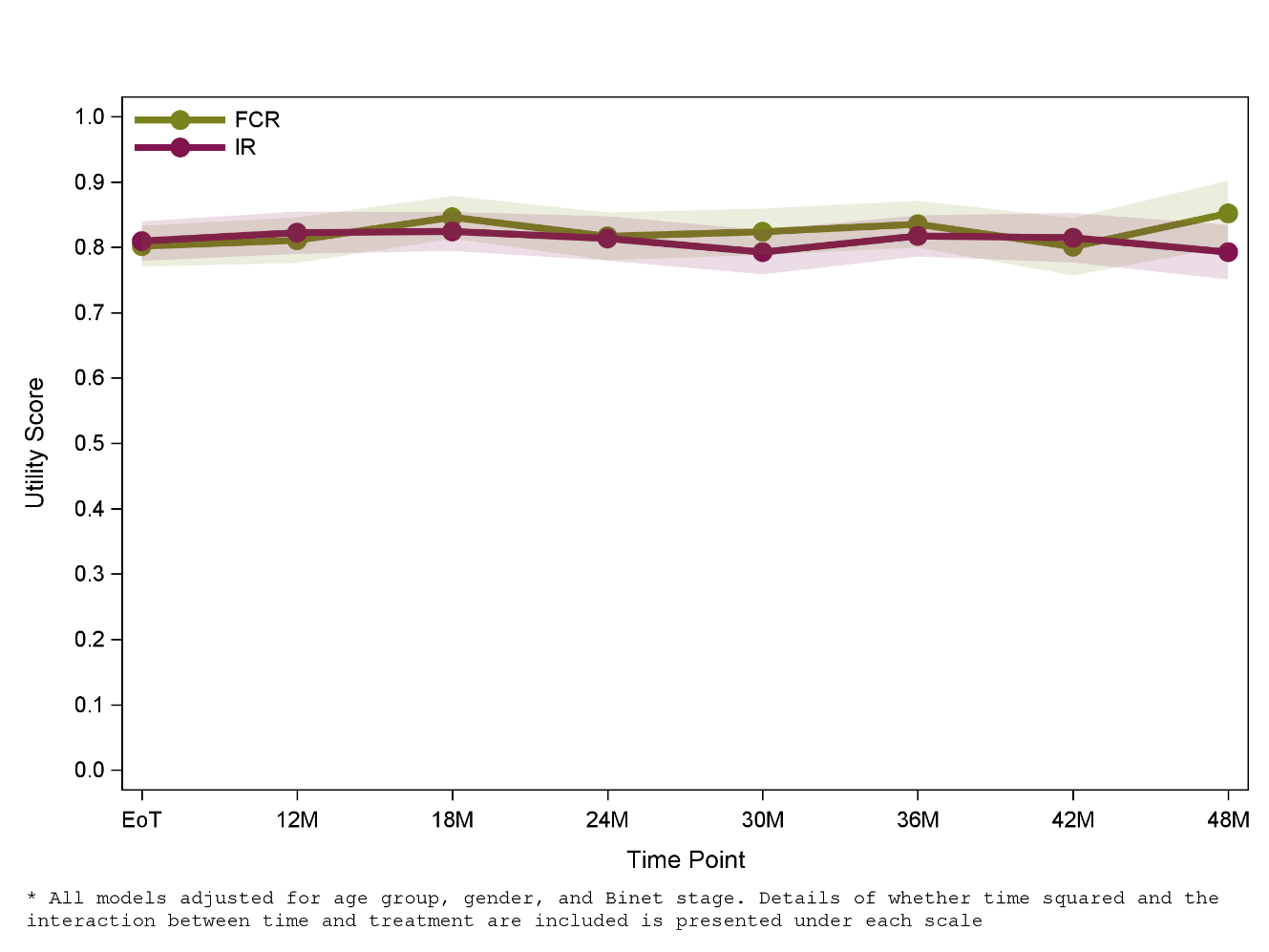
**

**
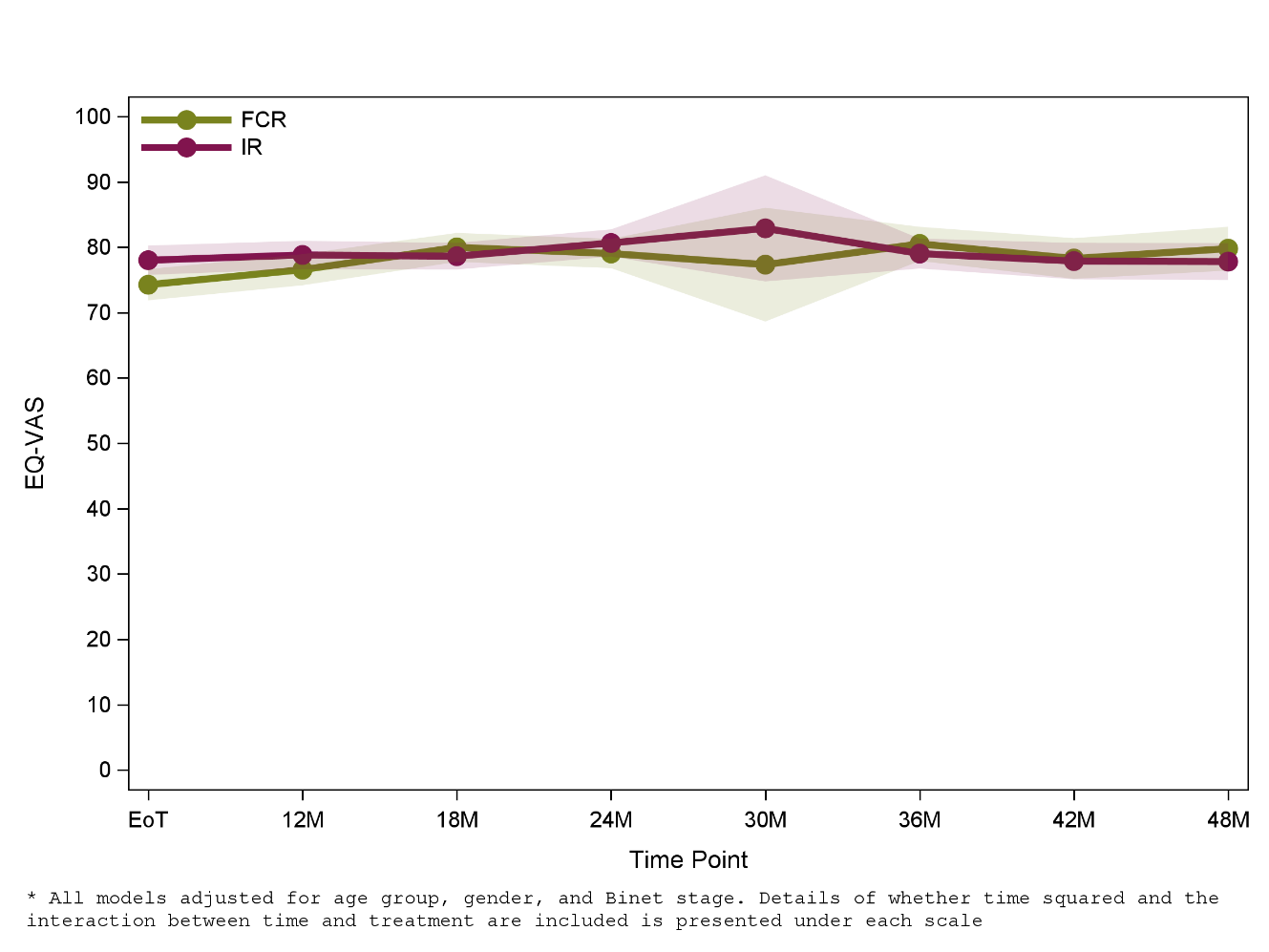
**

**(b)**

**
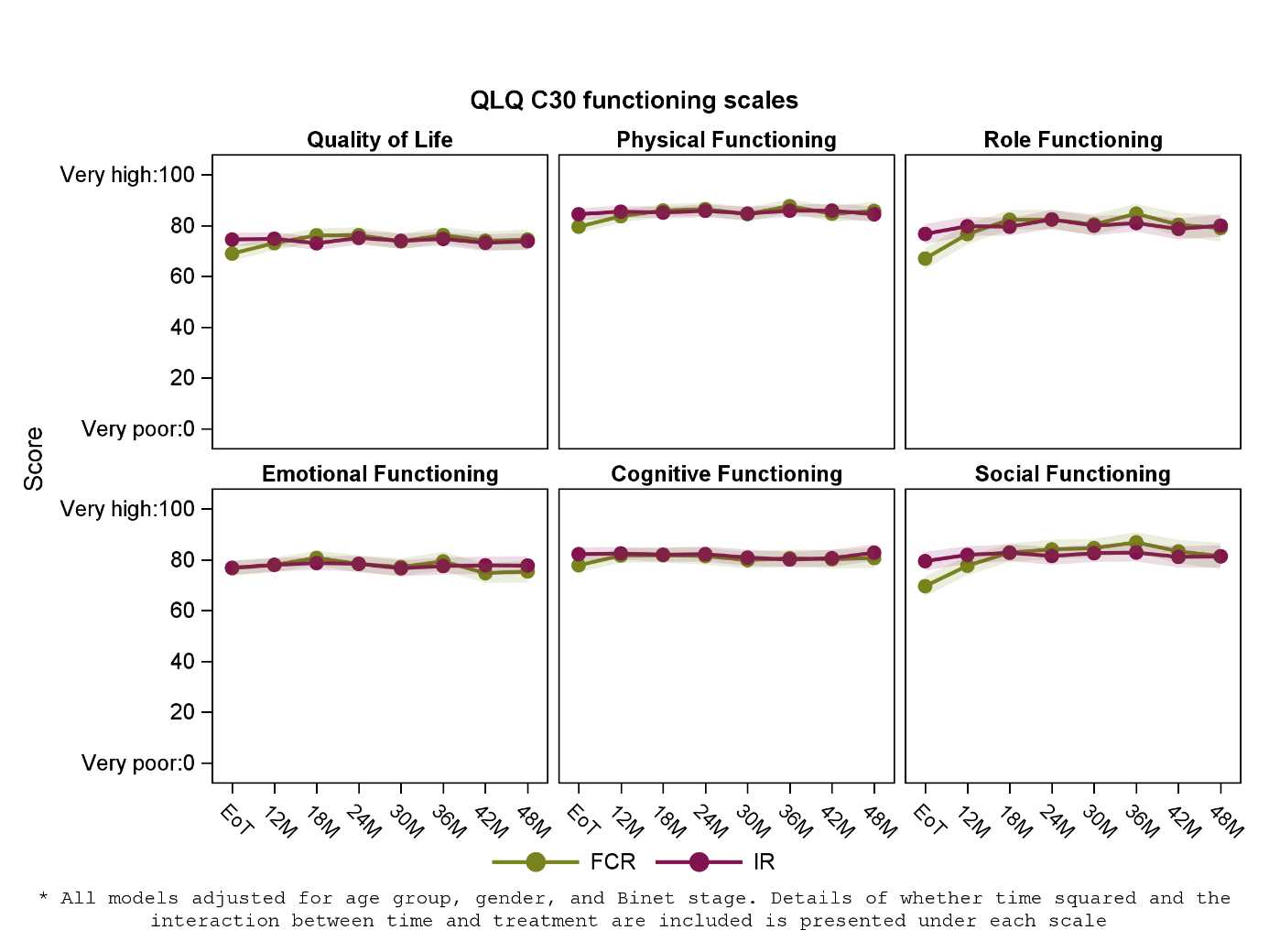
**

**(c)**

**
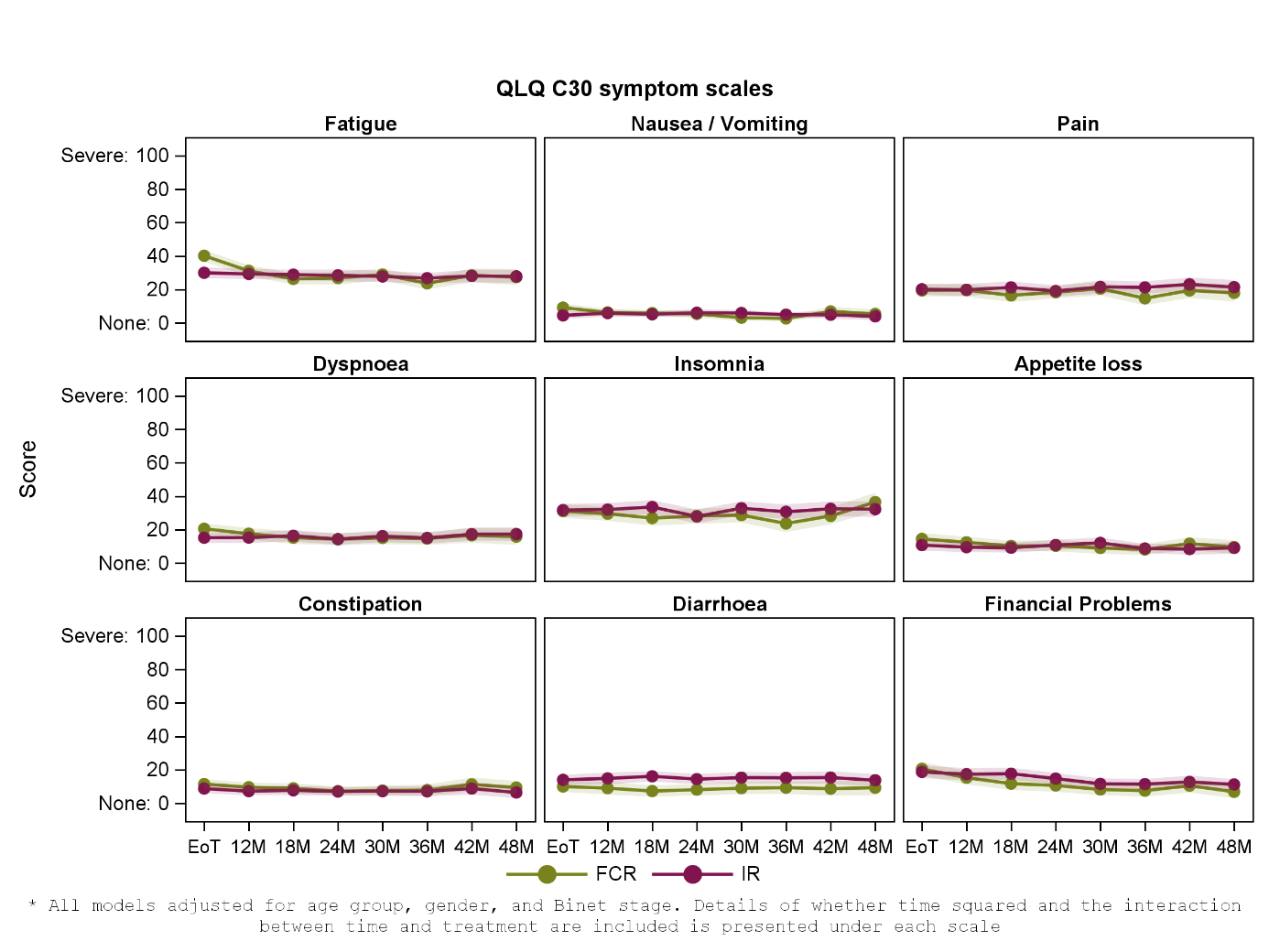
**

**(d)**

**
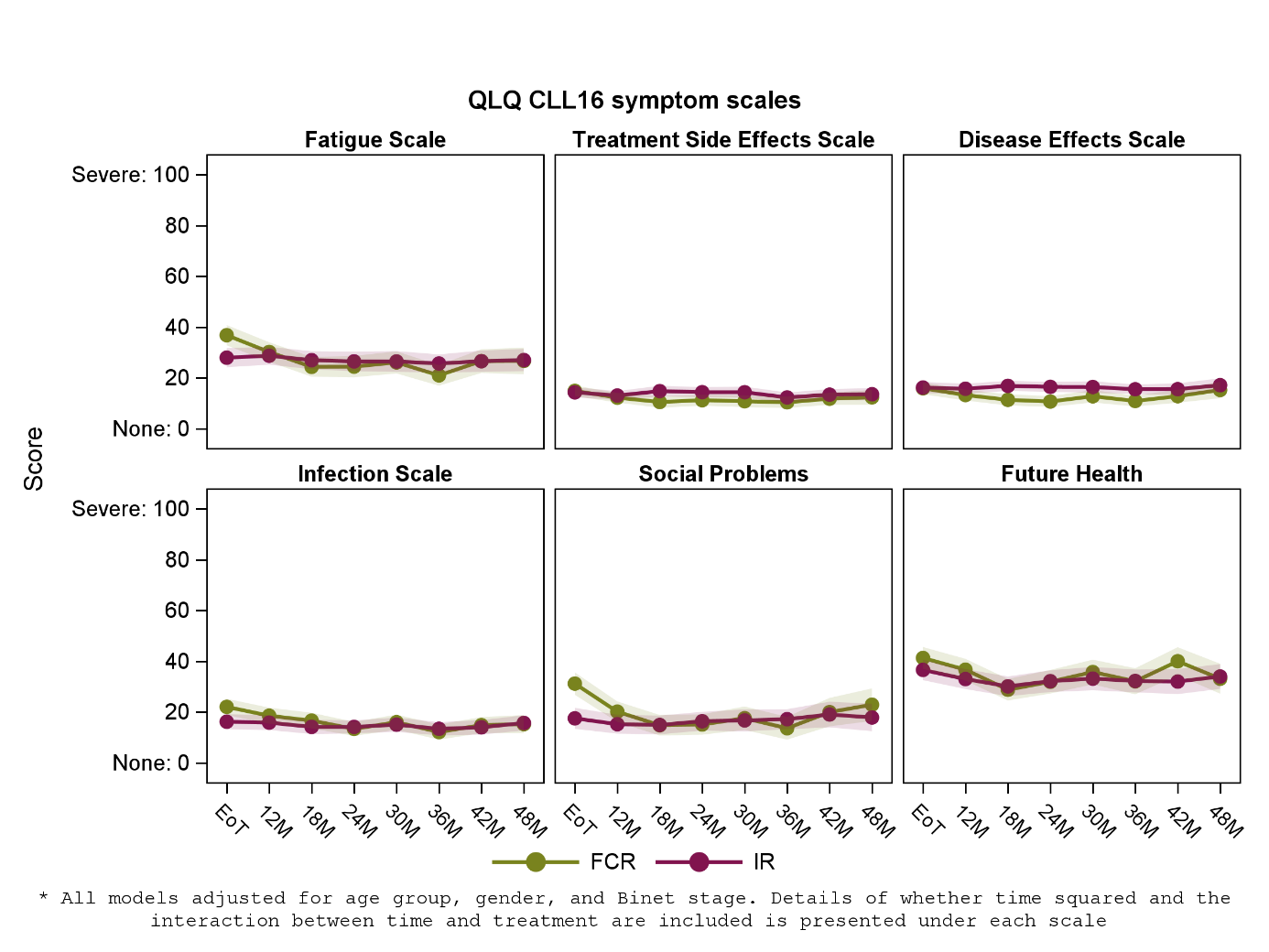
**

EORTC, European Organisation for Research and Treatment of Cancer; QLQ-C30, Quality of Life C30 Questionnaire; QLQ-CLL16, QLQ CLL Module; GHS–QoL=global health status–quality of life; IR, ibrutinib and rituximab; FCR, fludarabine, cyclophosphamide and rituximab

## Figure S4: Mean scores adjusted for baseline in participants aged greater than 65 years for (a) EQ-5D Utility Index and VAS, (b) EORTC QLQ C30 Functioning Scales, (c) EORTC QLQ C30 Symptom Scales and (d) EORTC QLQ CLL16 Symptom Scales

**(a)**

**
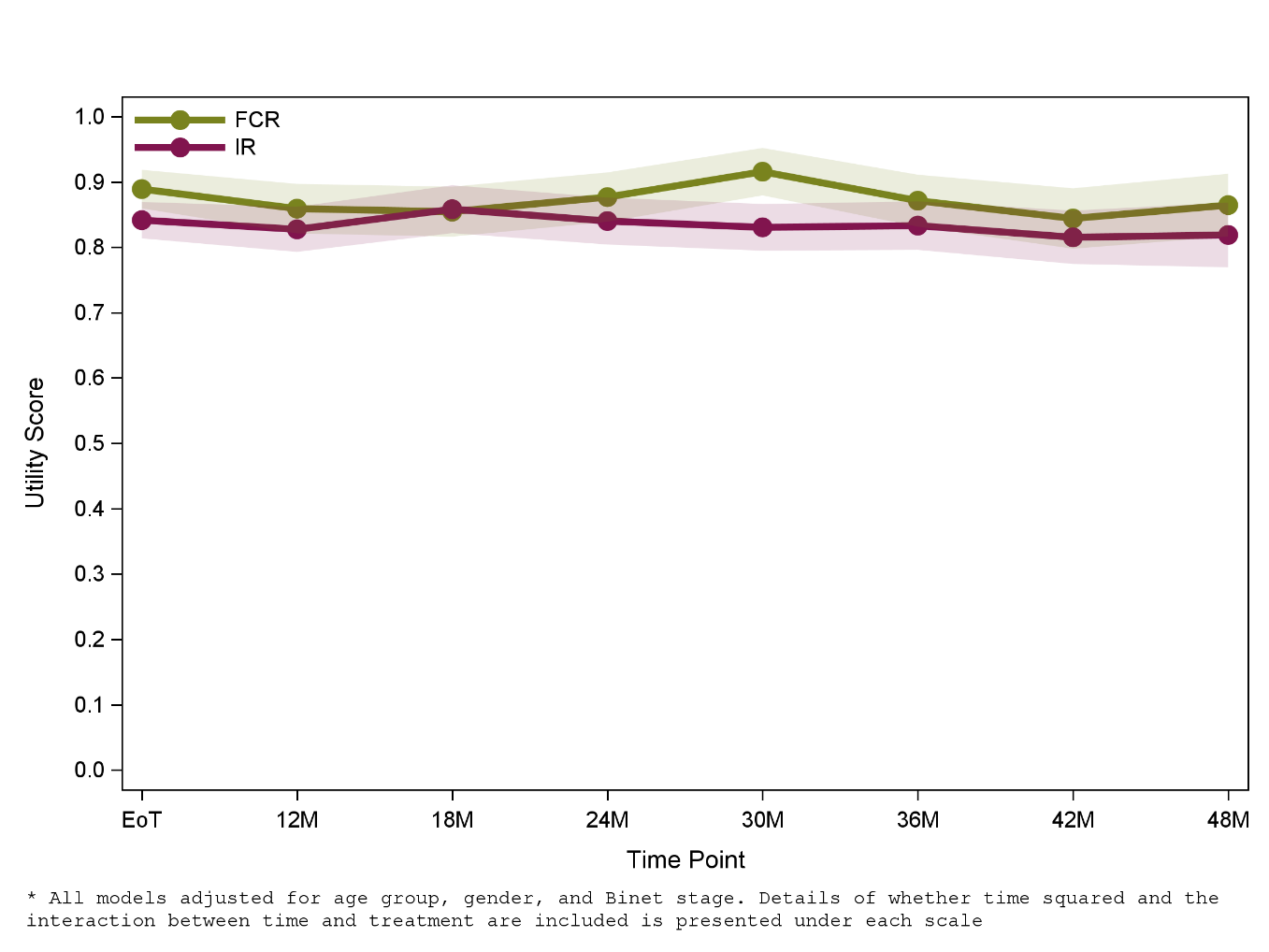
**

**
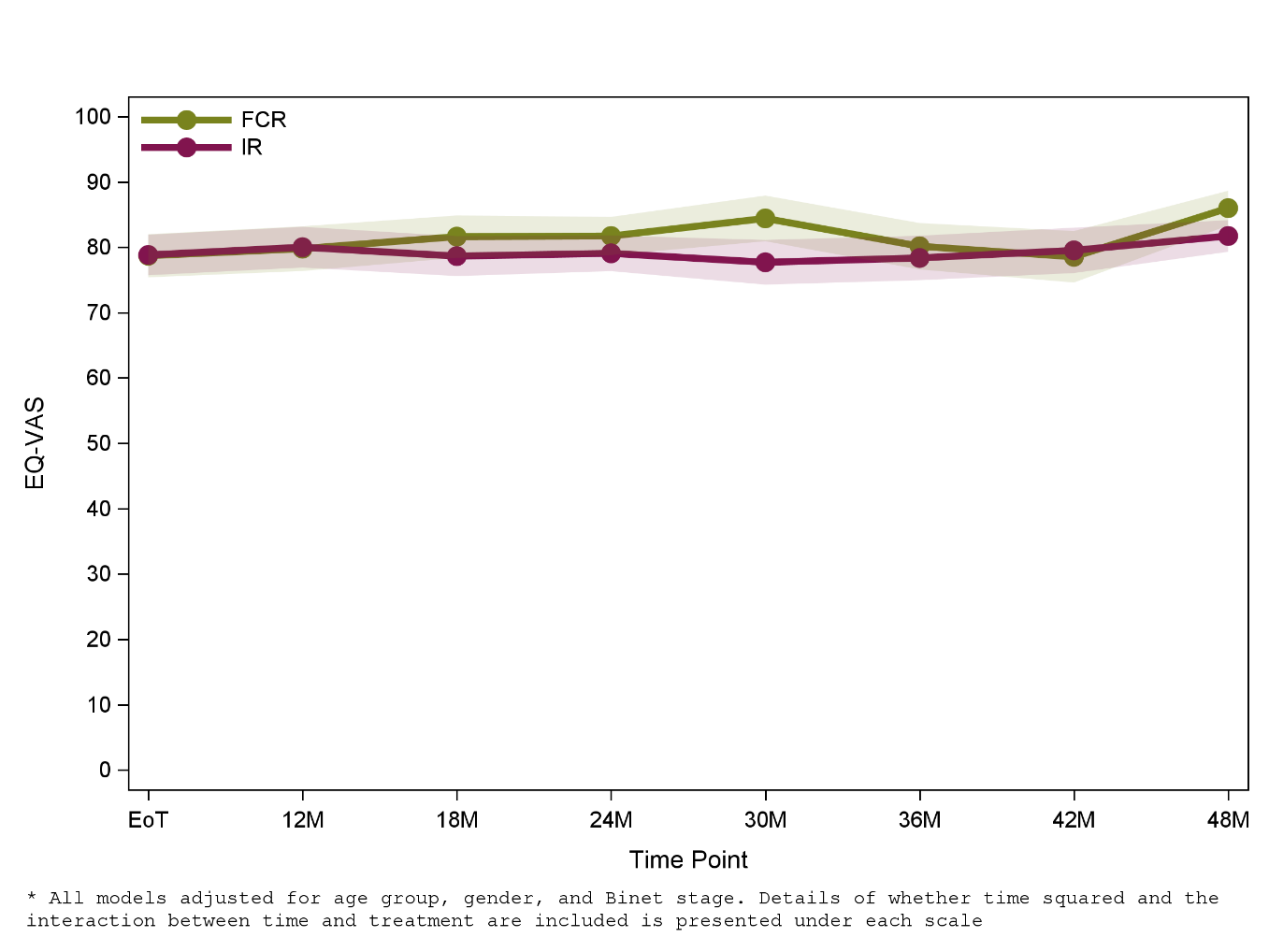
**

**(b)**

**
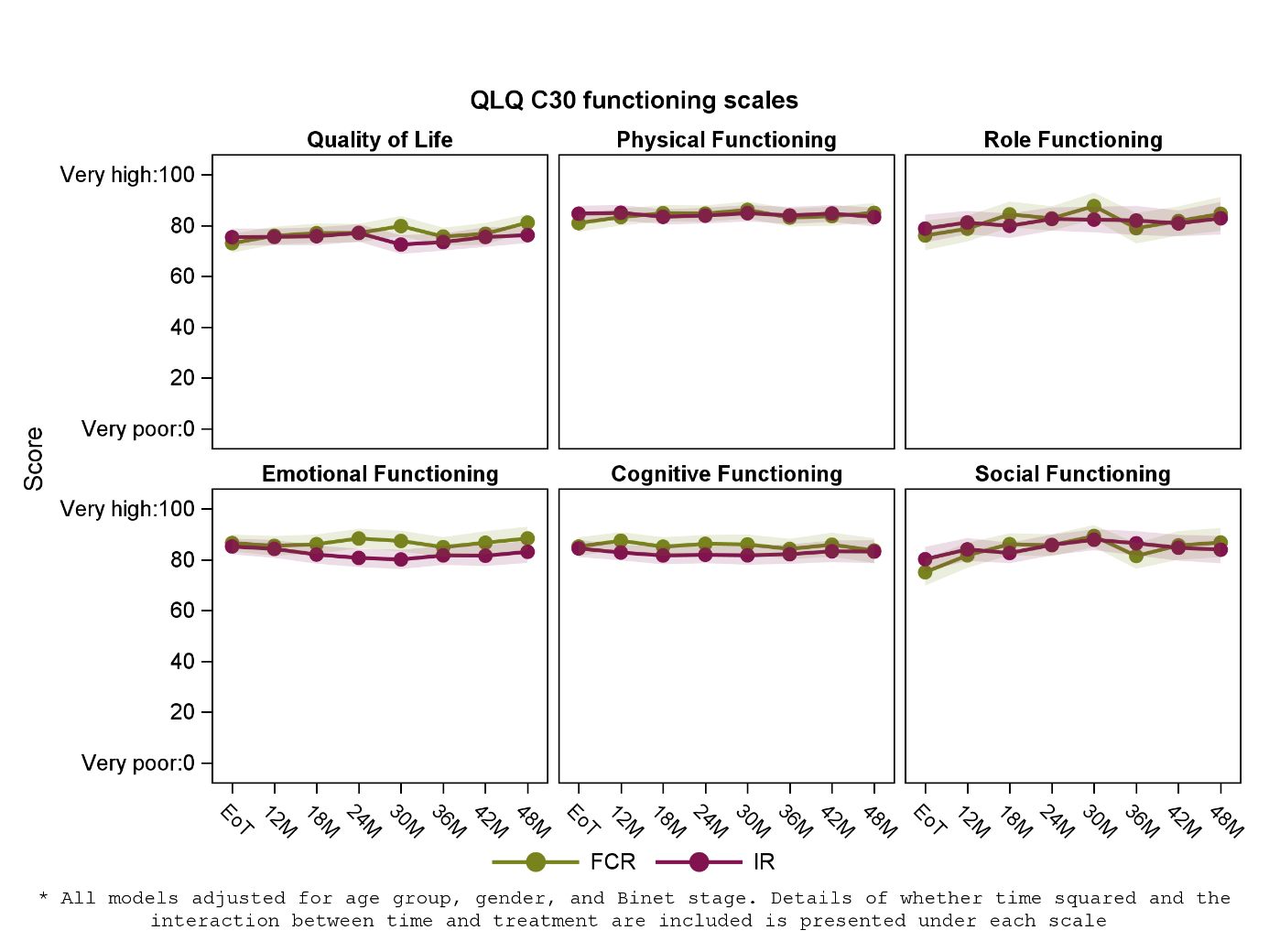
**

**(c)**

**
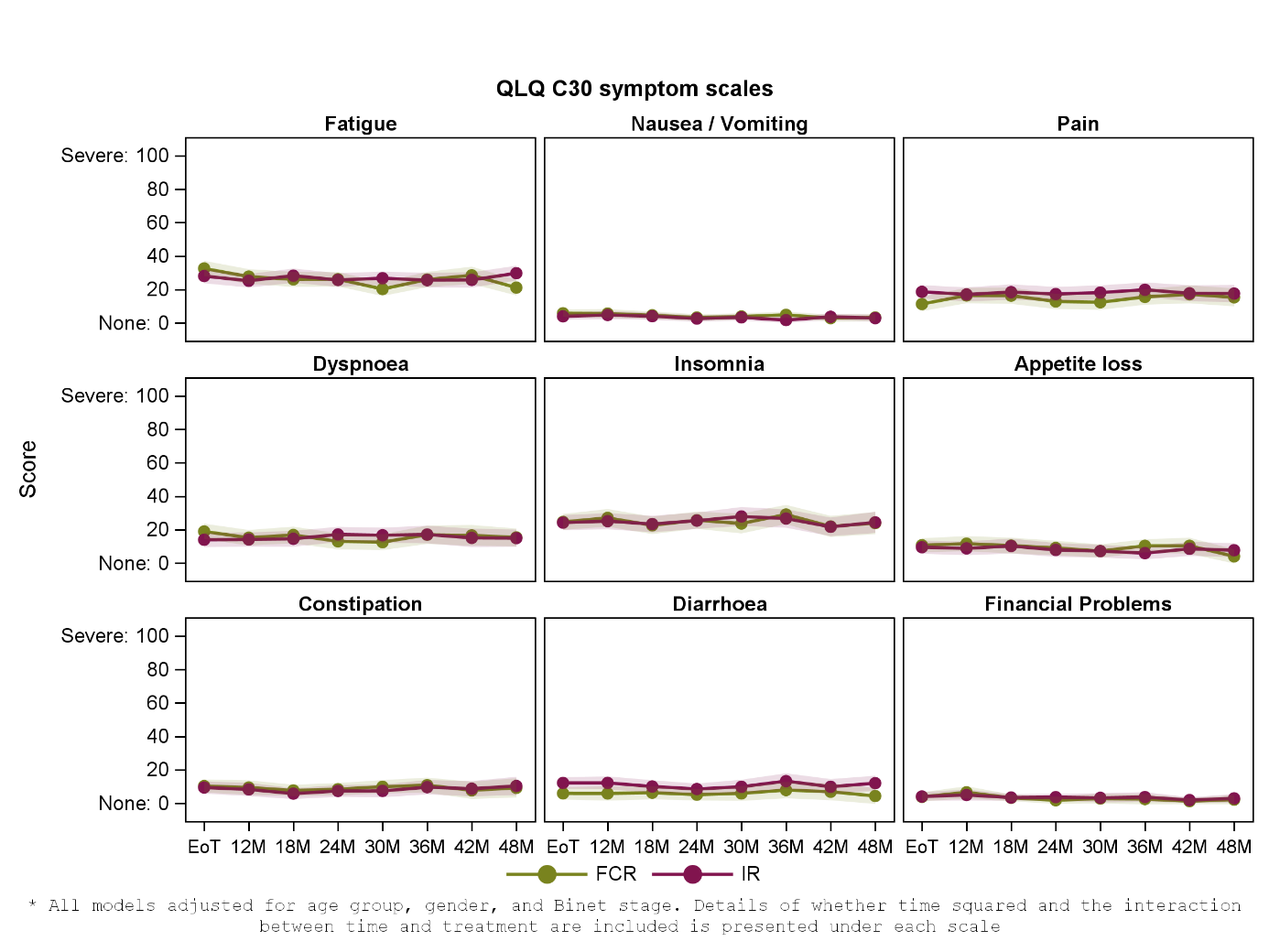
**

**(d)**

**
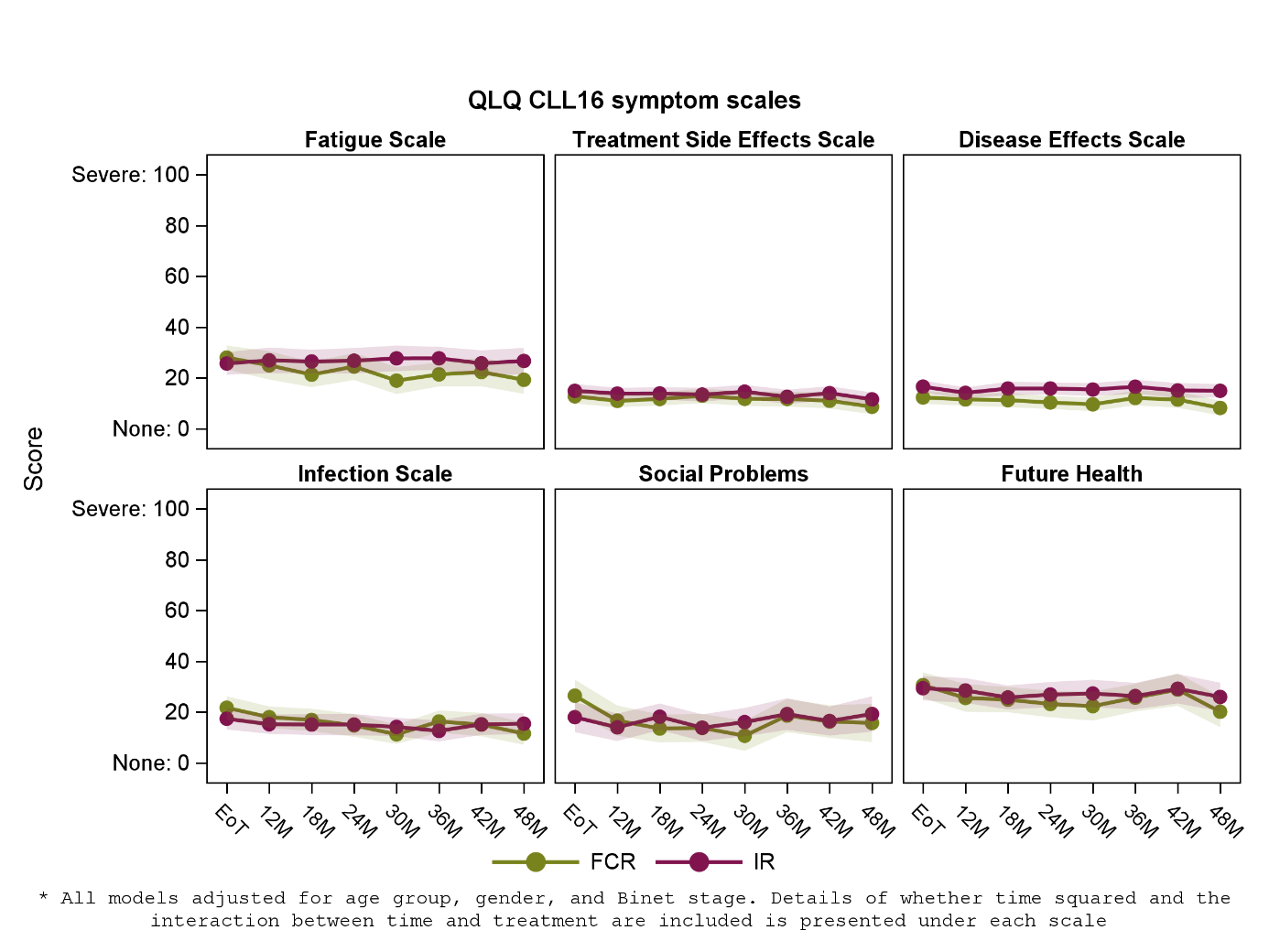
**

EORTC, European Organisation for Research and Treatment of Cancer; QLQ-C30, Quality of Life C30 Questionnaire; QLQ-CLL16, QLQ CLL Module; GHS–QoL=global health status–quality of life; IR, ibrutinib and rituximab; FCR, fludarabine, cyclophosphamide and rituximab
